# Unlocking the diagnostic power of plasma extracellular vesicle miR-200 family in pancreatic ductal adenocarcinoma

**DOI:** 10.1186/s13046-024-03090-z

**Published:** 2024-07-08

**Authors:** Daniel S.K. Liu, Jisce R. Puik, Bhavik Y. Patel, Morten T. Venø, Mahrou Vahabi, Mireia Mato Prado, Jason P. Webber, Eleanor Rees, Flora M. Upton, Kate Bennett, Catherine Blaker, Benoit Immordino, Annalisa Comandatore, Luca Morelli, Shivan Sivakumar, Rutger-Jan Swijnenburg, Marc G. Besselink, Long R. Jiao, Geert Kazemier, Elisa Giovannetti, Jonathan Krell, Adam E. Frampton

**Affiliations:** 1 Department of Surgery & Cancer, Imperial College London, Hammersmith Hospital, Du Cane Road, W120HS, London, UK; 2 Department of Surgery, Amsterdam UMC Location Vrije Universiteit Amsterdam, De Boelelaan 1117, Amsterdam, The Netherlands; 3 Cancer Center Amsterdam, Imaging and Biomarkers, Amsterdam, The Netherlands; 4 Department of Clinical and Experimental Medicine, Faculty of Health and Medical Sciences, The Leggett Building, University of Surrey, Guildford, Surrey GU2 7WG UK; 5 HPB Surgical Unit, Royal Surrey County Hospital, Guildford, Surrey UK; 6 Department of Molecular Biology and Genetics, Interdisciplinary Nanoscience Center, Aarhus University, 8000 Aarhus C, Aarhus, Denmark; 7 Omiics ApS, 8200 Aarhus N, Aarhus, Denmark; 8 UK Dementia Research Institute, Institute of Neurology, University College London, London, UK; 9 Institute of Life Science, Swansea University Medical School, Swansea University, Swansea, UK; 10 Institute of Life Sciences, Sant’Anna School of Advanced Studies, Pisa, 56127 Italy; 11 General Surgery Unit, Department of Translational Research and New Technologies in Medicine and Surgery, University of Pisa, Pisa, 56100 Italy; 12 Oncology Department, Institute of Immunology and Immunotherapy, Birmingham Medical School, University of Birmingham, Birmingham, B15 2TT UK; 13 Department of Surgery, Amsterdam UMC Location University of Amsterdam, Meibergdreef 9, Amsterdam, The Netherlands; 14 Cancer Pharmacology Lab, Fondazione Pisana per la Scienza, San Giuliano, Pisa 56016 Italy; 15 Laboratory of Medical Oncology, Amsterdam UMC Location Vrije Universiteit Amsterdam, Amsterdam, The Netherlands

**Keywords:** microRNAs, Extracellular vesicles, Biomarkers, Pancreatic ductal adenocarcinoma

## Abstract

**Background:**

Distinguishing benign from malignant pancreaticobiliary disease is challenging because of the absence of reliable biomarkers. Circulating extracellular vesicles (EVs) have emerged as functional mediators between cells. Their cargos, including microRNAs (miRNAs), are increasingly acknowledged as an important source of potential biomarkers. This multicentric, prospective study aimed to establish a diagnostic plasma EV-derived miRNA signature to discriminate pancreatic ductal adenocarcinoma (PDAC) from benign pancreaticobiliary disease.

**Methods:**

Plasma EVs were isolated using size exclusion chromatography (SEC) and characterised using nanoparticle tracking analysis, electron microscopy and Western blotting. EV-RNAs underwent small RNA sequencing to discover differentially expressed markers for PDAC (*n* = 10 benign vs. 10 PDAC). Candidate EV-miRNAs were then validated in a cohort of 61 patients (*n* = 31 benign vs. 30 PDAC) by RT-qPCR. Logistic regression and optimal thresholds (Youden Index) were used to develop an EV-miR-200 family model to detect cancer. This model was tested in an independent cohort of 95 patients (*n* = 30 benign, 33 PDAC, and 32 cholangiocarcinoma).

**Results:**

Small RNA sequencing and RT-qPCR showed that EV-miR-200 family members were significantly overexpressed in PDAC vs. benign disease. Combined expression of the EV-miR-200 family showed an AUC of 0.823. In an independent validation cohort, application of this model showed a sensitivity, specificity and AUC of 100%, 88%, and 0.97, respectively, for diagnosing PDAC.

**Conclusions:**

This is the first study to validate plasma EV-miR-200 members as a clinically-useful diagnostic biomarker for PDAC. Further validation in larger cohorts and clinical trials is essential. These findings also suggest the potential utility in monitoring response and/or recurrence.

**Graphical Abstract:**

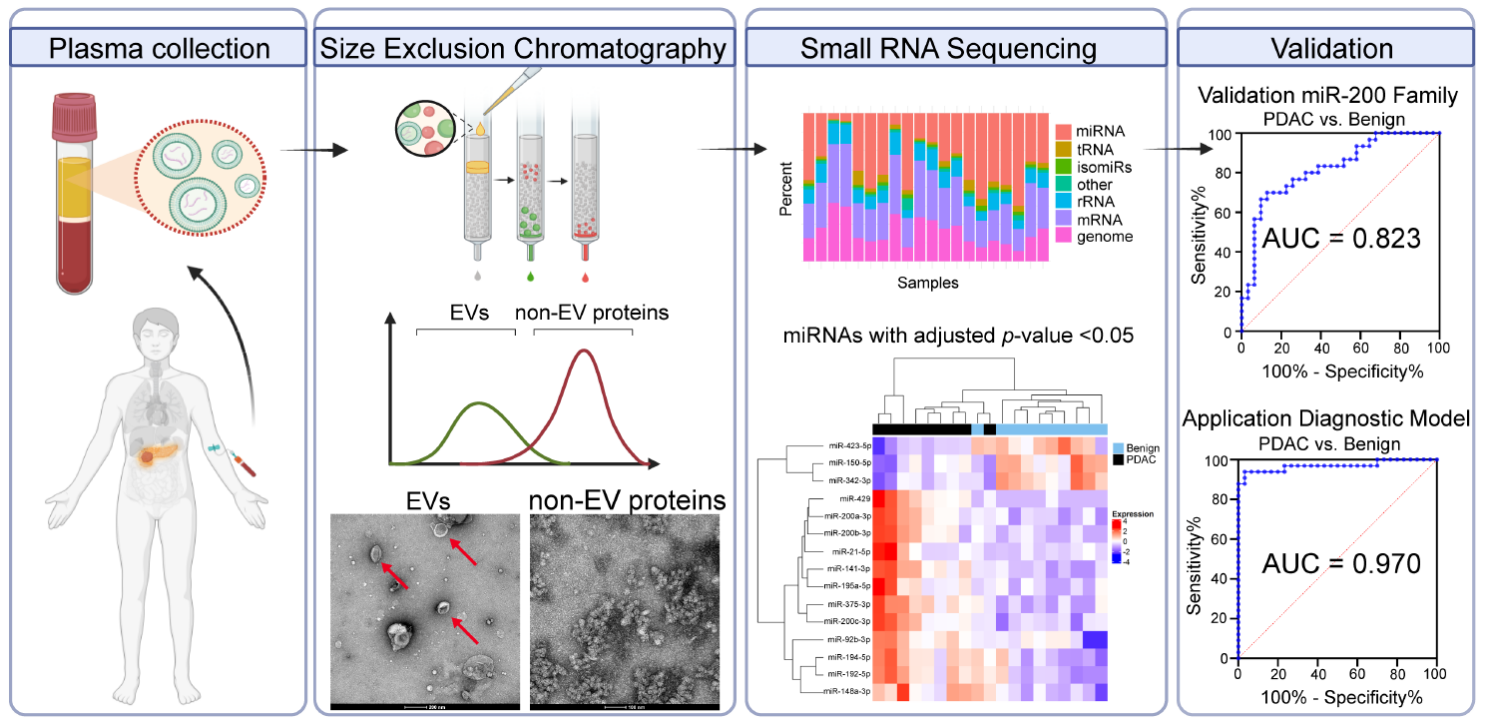

**Supplementary Information:**

The online version contains supplementary material available at 10.1186/s13046-024-03090-z.

## Background

Pancreatic ductal adenocarcinoma (PDAC) is a highly lethal disease with little recent improvement in the 5-year survival rate, which remains at approximately 13% [1, 2]. Due to its aggressive nature and a lack of specific symptoms, patients are often diagnosed at a late stage and therefore few patients are eligible for surgical resection. Even after surgery and chemotherapy, the prognosis remains around 20% after 5-years [1, 2]. An improved understanding of the molecular landscape of PDAC will allow further development of personalised (neo)adjuvant therapies to help improve survival [3]. Accurate diagnosis allows patients to be selected for prompt neoadjuvant treatment, and prevent futile surgical intervention in patients with benign disease. However, several malignant and benign pancreaticobiliary diseases share key features with PDAC, and current diagnostic tests for PDAC lack sensitivity and specificity [4]. Novel biomarkers are required to ensure accurate diagnosis, assess disease burden and predict which patients will benefit from chemotherapy and/or surgery. Extracellular vesicles (EVs) are increasingly being recognised as a source of potential biomarkers as they are naturally secreted by all cell types, including tumour cells, and contain cargo such as small and large RNAs [5]. This study aimed to establish a diagnostic plasma EV-derived microRNA (miRNA) signature to discriminate PDAC from benign pancreaticobiliary disease, and to verify its diagnostic value for clinical application.

Currently, the only used biomarker in blood testing for PDAC is CA 19 − 9 [6]. Due to insufficient sensitivity and specificity, CA 19 − 9 cannot reliably distinguish between PDAC and benign pancreaticobiliary disease [6]. Serum levels can be raised in patients with jaundice or other benign inflammatory pancreatic disorders [7], and CA 19 − 9 is undetectable in patients who are Lewis antigen negative (7–10% of the population) [8]. Therefore, CA 19 − 9 has a limited role in diagnosis, and is mostly used to monitor response to systemic treatment.

EVs are phospholipid bilayer enveloped structures that have been shown to facilitate intercellular communication [9]. They selectively contain RNA alongside other cargo, such as DNA, proteins and lipids, and transfer such content between cells [9]. This can lead to phenotypic changes in recipient cells, and as such, EVs modulate many biological processes and play an important role in tumorigenesis and metastasis [5]. Many techniques have been developed to improve the yield and purity of EV isolation [10]. Whilst ultracentrifugation (UC) remains the most popular technique for the extraction of EVs from fluid samples, we have developed a protocol for EV isolation using size exclusion chromatography (SEC) which is fast and uses low sample input volumes [11]. Employed as a chromatographic technique since the 1990s, SEC has not previously been recognised for the isolation of plasma-derived EVs usable in next-generation sequencing methods. The increasing recognition of SEC in biomarker research is attributed to its prospective clinical applicability and the possibility of standardization [12, 13]. Several reports have shown that blood-derived EVs from patients with PDAC contain miRNAs with differential expression [14–18]. However, these studies have marked differences in their methodology, including miRNA target selection, EV isolation technique, miRNA profiling, negative control groups, sample size, and methods of validation.

MicroRNAs are short (∼17–25 nucleotides) non-coding RNAs that function as regulators of gene expression at a post-transcriptional level [19]. MiRNAs have been shown to play a role in each of the hallmarks of cancer and can function in an oncogenic or tumour suppressive manner [20]. When bound to Argonaute 2 protein or within EVs, miRNAs have shown high resistance to endonuclease activity and are stably expressed in both tissue and the systemic circulation [21]. As such, miRNAs form an appealing target for circulating biomarker research [22, 23]. Many studies have identified circulating cell-free miRNA signatures for cancer [24], including PDAC [25]. However, no previous study has applied small RNA sequencing on RNA from SEC-isolated plasma EVs for biomarker discovery. This multicentric study reports on the characterisation of SEC-isolated plasma EVs, the results of small RNA sequencing from these EVs to discover differentially expressed miRNAs in PDAC, and the validated signature, both in a technical validation cohort, and a second validation cohort.

## Methods

### Study design

**Fig. 1:**
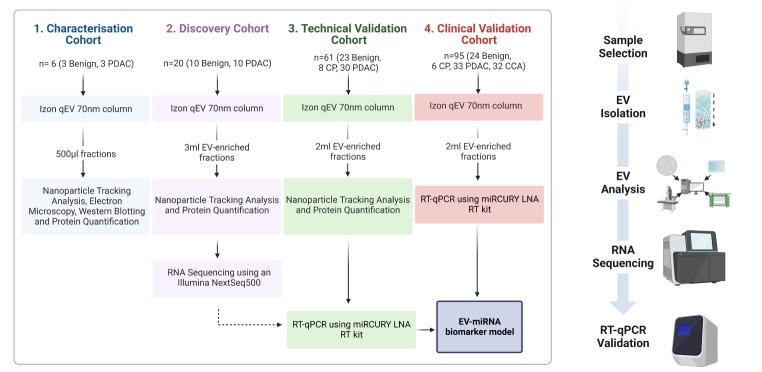
Study Design. The phases of our study were divided into 4 broad stages which can be used in the future for any EV biomarker study. Characterisation of EVs isolated by SEC were analysed for the presence of EVs and EV surface proteins. The discovery of EV-associated miRNAs was undertaken by next-generation small RNA sequencing, and this was validated with RT-qPCR to confirm differential expression. This data was used to inform a diagnostic EV-miRNA model, which was then applied to an individual cohort of patients

This study consisted of four stages: (1) characterisation of SEC-isolated plasma EVs (3 PDAC vs. 3 benign); (2) small RNA sequencing of plasma EV-RNAs to discover differentially expressed miRNAs between PDAC (*n* = 10) and benign pancreaticobiliary disease (*n* = 10); (3) validation of candidate miRNAs (*n* = 31 benign vs. 30 PDAC) using quantitative reverse transcription polymerase chain reaction (RT-qPCR); and (4) test the applicability of the best miRNA model in an independent validation cohort (*n* = 33 PDAC, 32 CCA and 30 benign). The study design is shown in Fig. 1. The primary endpoint was discovery and validation of a plasma EV-miRNA model able to detect PDAC vs. benign pancreaticobiliary disease. Plasma samples from patients with cholangiocarcinoma (CCA) were added as a positive control group to our second validation, allowing for the evaluation of the biomarker in a cancer distinct from PDAC. Additionally, for purposes of investigation, plasma samples from healthy donors (*n* = 14) were included.

Blood samples and clinicopathological data were collected prospectively. Samples were obtained from patients who attended the endoscopy unit at Imperial College NHS Trust (Hammersmith Hospital, London, UK) between November 2017 and March 2020 for biliary drainage. Ethical approval was obtained from Imperial College Healthcare Tissue Bank for the collection and storage and analysis of patient samples (SUR_AF_17_044, “Molecular Detection and Stratification of HPB Cancers”) and the ethical board of the University of Pisa (protocol#23,744). Permission for blood sampling from healthy/asymptomatic control subjects was granted by the local institutional review boards. These healthy control subjects signed an informed consent and some of the samples analysed have been featured in previously published studies [26, 27]. All investigations have been performed in accordance with the principles of the Declaration of Helsinki. Written informed consent was obtained from all participants included in this study.

### Extracellular vesicle isolation from peripheral blood

Patients were fasted from midnight the previous day according to the protocol for patients attending ERCP with sedation. These peripheral fasting blood samples were drawn via a freshly inserted 18-22G peripheral cannula or 21-23G butterfly syringe into BD K2 Ethylenediaminetetraacetic acid (EDTA) lined tubes and processed within 4 h. Blood was centrifuged at 2,500 x *g* for 10 min at room temperature to remove cells and aliquot the resulting platelet-free supernatant (plasma). The latter was snap-frozen at -80 °C until required (see reporting form in Additional File 2: MIBlood-EV Standardized Reporting Tool for Blood EV Research Human). For EV isolation, 1 mL of plasma was thawed on ice, after which SEC was performed using commercially available qEVoriginal 70 nm SEC columns (iZON Science) according to the manufacturer’s protocol. Plasma samples were loaded onto the column and eluted with PBS (pH 7.4; Sterile-filtered, Sigma Aldrich). The eluate was collected in 30 sequential fractions of 0.5 mL, which were used immediately for EV characterisation and RNA extraction, or frozen at -80 °C until required. For the purposes of analysis, we deemed fractions 7–10 as EV-enriched (EV) and 22–24 as free protein-enriched (PROT) after initial characterisation.

### Pathology data collection

The disease aetiology was determined based on diagnostic histopathology from surgically resected specimens or biopsy specimens (from open or laparoscopic surgery, percutaneous needle, or endoscopic biopsy) and in the case of benign disease, cytology/histopathology, and clinical criteria after ≥ 12 months of follow-up. Aetiology and clinical staging were determined after a multidisciplinary team (MDT) meeting consisting of at least a consultant hepatopancreaticobiliary surgeon, consultant hepatopancreaticobiliary physicians, consultant histopathologists, consultant radiologists and cancer nurse specialists. ERCP findings, endoscopic ultrasound (EUS) findings, biliary brushing cytology, and fine needle aspiration cytology were anonymised and recorded. Benign diseases included chronic pancreatitis, benign IPMN and choledocholithiasis.

### Nanoparticle tracking analysis

Nanoparticle tracking analysis (NTA) was performed using a ZetaView® PMX-120 (Particle Metrix, Germany) to determine particle concentration and size distribution down to a limit of 60 nm. Polystyrene microspheres (100 nm) were used to configure and calibrate the instrument according to the manufacturer’s protocol. Fractions were diluted 50 − 10,000-fold in PBS to ensure a number of particles per position between 50 and 200 particles. For each measurement, 11 positions were scanned and 30 frames per position captured. Videos were analysed by the in-built ZetaView Software 8.05.04, Camera 0.712 μm/pixel, with the following analysis parameters: Maximum area: 1000, Minimum area: 10; Minimum brightness; 30. Outlier measurements were automatically detected using the software’s Grubb’s test and excluded from the analyses. The inclusion of at least 9 camera positions was deemed a satisfactory measurement.

### Protein quantification

A Bicinchoninic Acid Protein Assay Kit (Thermo Scientific™ Pierce™) was used to quantify the protein concentration in each sample. The assay was performed according to the manufacturer’s microplate protocol. Briefly, triplicate volumes of 1–25 µL was used and added to 200 µL of Working Reagent in a Corning® Costar® TC-Treated 96-Well Plate. The plate was mixed thoroughly for 30 s and incubated for 30 min at 37 °C. Absorbance was measured at 562 nm using an Optimax Tunable Microplate Reader and a standard curve prepared bovine serum albumin (2.5–2000 ng/µL) according to the manufacturer’s protocol. Measurements below 20 µg/mL (limit of assay) were not included in the analysis.

### Western blotting

Samples for analysis were concentrated using a Vivaspin Turbo 4 (10 kDA MW cut-off) by spinning samples at 4,000 x *g* for 2 h at 4 °C. Samples were then lysed on ice with 10X RIPA Buffer with protease inhibitor phenylmethylsulfonyl fluoride. Lysed protein was quantified and equal amounts of protein (5–20 µg of total protein) of each sample were prepared with Pierce™ LDS 4X Sample Buffer (Thermo Scientific) and a reducing agent, dithiothreitol, if required. Samples were heated at 95 °C for 5 min, separated on 11% sodium dodecyl sulfate polyacrylamide gel electrophoresis (SDS-PAGE) gels and transferred onto a nitrocellulose membrane. Total protein bands were detected using Ponceau S solution or No-Stain™ Protein Labelling Reagent (Invitrogen™). Membranes were blocked with 5% non-fat powdered milk in Tris-buffered Saline, 0.1% Tween-20 and incubated with primary antibodies overnight at 4 °C. Membranes were incubated with appropriate HRP-conjugated IgG secondary antibodies (1:2500) for 1 h at room temperature. Protein expression was visualised after incubation with Immobilon™ Western Chemiluminescence HRP Substrate on an iBright 1500 (Thermo Fisher). Images were analysed with Image Lab Software 6.0.1 (Bio-Rad Laboratories, USA) and membranes then stripped for repeat immunoblotting by incubating with Restore™ Plus Western Blot Stripping Buffer (#46,430, Thermo Fisher Scientific, USA).

### Transmission electron microscopy

All preparation and imaging were performed at the Electron Microscopy Centre, South Kensington Campus, Imperial College London. In brief, 8 µl samples were deposited onto 200–300 mesh glow-discharged copper grids for 2 min at room temperature, washed twice with MilliQ water and then negatively stained with 2% uranyl acetate. The grids were observed using a Tecnai™ T12 TWIN (FEI, USA) transmission electron microscope at magnification ranges 4,000-120,000x.

### Next-generation small RNA sequencing

Samples were prepared for small RNA sequencing using Qiagen’s QIAseq small RNA Library Prep kit. The finished libraries were quality controlled using an Agilent Bioanalyzer 2100 and quantified by use of qPCR. Libraries were pooled and sequenced on an Illumina NextSeq500 sequencer by single-end 75 base pair (bp) sequencing. The raw data was quality filtered and trimmed by fastx_toolkit, and adaptor sequences were removed using Cutadapt. Unmapped reads were mapped to miRNAs from miRBase v22 allowing zero mismatches but allowing for non-templated 3’ A and T bases. Quality control was performed using FastQC to ensure high quality data. The miRNA read counts were subjected to differential expression analysis and normalization using DESeq2 in R.

### Quantitative reverse transcription polymerase chain reaction

Reverse transcription of RNA samples was undertaken using the miRCURY LNA RT kit (QIAGEN) for detection of low RNA inputs without preamplification. A volume of 1 µL Total RNA was mixed by volume with 1 µL of RT Probe Reaction Buffer (according to manufacturer’s protocol, modified to use 5 µL volume), 0.25 µL of 10X miRCURY RT Enzyme Mix, 0.25 µL of UniSp6 RNA (108 copies/µL) spike-in and 2.5 µL of RNase-free water in a 48-well PCR plate. Samples were incubated in a thermal cycler at 40 °C for 60 min to allow primer annealing/elongation, 5 min at 85 °C to inactivate the reverse transcriptase and stored at 4 °C.

Quantitative PCR reactions were performed in triplicate using the miRCURY LNA SYBR Green PCR Kit (QIAGEN) following manufacturer’s instructions. In brief, cDNA samples were diluted 1:30 with RNase-free water and 3 µL was then added to 5 µL of 2X SYBR Green Master Mix, 0.5 µL of ROX Reference Dye, 1 µL of miRCURY LNA miRNA-specific PCR assay and 0.5 µL of RNase free water in a MicroAmp Fast Optical 96-well Reaction plate (Thermo Fisher Scientific). This was sealed using a MicroAmp Optical Adhesive Film and run through miRCURY LNA specific PCR cycling conditions on a Real-Time PCR System. This was a ‘hot-start’ protocol involved an initial step to denature the cDNA at 95 °C for 2 min, followed by 40 cycles of denaturation at 95 °C for 10 s and annealing/elongation at 56 °C for 60 s. Measurement of fluorescence was undertaken after each cycle and the threshold cycle (Ct) determined by the built-in software. Ct values that were discordant (> 0.5 apart) were repeated and the average used for further analysis. MiRNAs with a Ct value > 40 were deemed not detected and set at 40 for calculation of relative expression.

### Calculation of relative expression

Reference genes for normalisation were determined using the NormFinder package for R (Version 5 January 2015) and small RNA sequencing data. Expression of the most stable miRNAs was determined again using RT-qPCR and analysed using NormFinder package for Microsoft Excel (Version 20, May 2010). The “stability value” is briefly summarised as a number which reflects the intra- and inter-group variances of an expressed gene with the lowest number identifying the gene with the lowest overall variance and inter-group difference. The miRNA pair with highest stability was identified using NormFinder algorithms on the RNA-sequencing data and compared for RT-qPCR expression in a subset of samples (*n* = 4). The geometric mean (the nth root of the product of n numbers) of these values together with expression of an exogenous spike-in (UniSp6) was obtained, defined as the EndoMean. In each sample, expression levels of target miRNAs were calculated using the comparative (ΔΔCt) method: 2^-(∆∆Ct), where ∆∆Ct = ∆Ct – mean ∆Ct control group, and ∆Ct = Ct EndoMean – mean Ct target miRNA. These values were further binary logarithmically transformed and shown as log fold expression.

### Statistical analysis

Statistical analyses were performed using GraphPad Prism 9.1.1. Receiver operating characteristic (ROC) curves were generated for each significantly deregulated EV-miRNA, leading to estimates of area under the curve (AUC) with 95% confidence intervals (CI). Multiple logistic regression was performed for candidates individually and combined using GraphPad Prism 9.1.1. Data was prepared as a binary outcome and all main effects were included in the model. Optimum cut-offs were determined using thresholds obtained from the ROC curve at the maximum Youden’s index, which is a single statistic that ranges from 0 to 1 and is determined by the formula (Specificity + Sensitivity − 1). Where multiple hypotheses were tested, an appropriate Benjamini–Hochberg (False Discovery Rate) correction was applied to give an adjusted p-value. An (adjusted) p-value of < 0.05 was considered statistically significant. To detect differences in clinicopathological variables between groups, the Mann–Whitney *U*-test was used for non-parametric data, the Student t-test for parametric data and the Fisher’s exact test for categorical data.

### Development of a diagnostic model and clinical validation

To test the applicability of a miRNA signature in an independent clinical validation cohort, a formula was generated by performing multiple logistic regression using ∆Ct RT-qPCR miRNA expression data from the technical validation cohort: *y* = β_0_ + β_1_∆Ct _1_ + … + β_n_∆Ct_n_. The formula outcome (*y*) was calculated by multiplying regression coefficients (β) of the formula with the measured miRNA expression (∆Ct). Youden’s index was used to determine the most optimal classification cut-off. In short, predicted probability values from logistic regression in the technical validation cohort were used as binary input (PDAC vs. benign) for ROC analysis, giving sensitivity and specificity at a number of thresholds. Then specificity + sensitivity – 1 was applied for all thresholds and the threshold with the highest outcome was determined the most optimal classification cut-off (i.e. at which the formula determined the predicted probability as PDAC or benign disease).

Next, the formula was applied to the ∆Ct RT-qPCR miRNA expression data of the clinical validation cohort, giving outcome *y* for each patient in the validation cohort. For classification, P = exp(*y*)/(1 + exp(*y*)) was applied to get a predicted probability value between 0 and 1. If the outcome was below the cut-off, the sample was determined as benign and if the outcome was above the cut-off, the sample was determined as malignant. Comparing the test results of the model with the true histopathological data led to the calculations for sensitivity, specificity negative predictive value (NPV) and positive predictive value (PPV). Predicted probabilities (P) were used for ROC analysis to generate an AUC value with CI for the model of a signature.

## Results

### Patients

Patient demographics are shown in Table 1. Within all cohorts, pre-therapeutic mean bilirubin and CA 19 − 9 levels were significantly raised in PDAC samples compared to the benign samples (*p* < 0.05). Tumour staging was predominantly T4 in both the discovery and technical validation cohort (100% and 67%, respectively), while in the clinical validation cohort staging was mostly T3 (46% of PDAC and 60% of CCA tumours). Most cancers causing biliary obstruction were anatomically located in the head of pancreas (81%).

**Table 1:** Clinicopathological patient characteristics

Patient characteristics		Discovery (*n* = 20)							Technical validation (*n* = 61)							Clinical validation (*n* = 95)							
	PDAC (*n* = 10)			Benign (*n* = 10)		*p*-value		PDAC (*n* = 30)			Benign (*n* = 31)		*p*-value		PDAC (*n* = 33)			CCA (*n* = 32)		Benign (*n* = 30)		*p*-value	
**Age, y (%)**																							
< 60	1		(10)	5	(50)			6		(20)	10	(33)			7		(21)	5	(16)	13	(43)		
≥ 60	9		(90)	5	(50)	0.1409		24		(80)	21	(67)	0.3844		26		(79)	27	(84)	17	(57)	**0.014**	
Mean (SD)	74		(12)	52	(16)	**0.0029**		69		(12)	66	(14)	0.4643		69		(11)	69	(8)	63	(13)	0.065	
**Sex (%)**																							
Female	3		(30)	6	(60)			9		(30)	9	(29)			15		(45)	15	(47)	11	(37)		
Male	7		(70)	4	(40)	0.3698		21		(70)	22	(71)	1.0000		18		(55)	17	(53)	19	(63)	0.504	
**History of PSC (%)**	0		(0)	1	(10)	1.0000		0		(0)	0	(0)	1.0000		1		(3)	0	(0)	0	(0)	1.000	
**Chronic pancreatitis (%)**	0		(0)	0	(0)	1.0000		2		(7)	8	(26)	0.0807		2		(6)	0	(0)	6	(20)	**0.011**	
**Blood parameters, mean (SD)**																							
CA 19 − 9 (U/mL)	4090		(5051)	19	(13)	**0.0040**		7371		(11,310)	303	(1025)	**0.0256**		951		(2310)	794	(2069)	28	(48)	**< 0.001**	
Bilirubin (ng/mL)	177		(103)	24	(12)	**< 0.0001**		229		(131)	51	(70)	**< 0.0001**		131		(184)	120	(141)	12	(9)	**< 0.001**	
CRP (µg/mL)	52		(90)	15	(14)	0.2370		57		(78)	28	(35)	0.1479		17		(22)	34	(48)	11	(18)	**0.039**	
**Anatomical location**																							
Head of pancreas	7		(70)					24		(80)					28		(85)	31	(97)				
Uncinate process	1		(10)					3		(10)					0		(0)	0	(0)				
Mixed HOP/Uncinate	2		(20)					3		(10)					0		(0)	0	(0)				
Body of pancreas	0		(0)					0		(0)					3		(9)	0	(0)				
Isthmus of pancreas	0		(0)					0		(0)					1		(3)	0	(0)				
Ampulla of vater	0		(0)					0		(0)					1		(3)	0	(0)				
Common bile duct	0		(0)					0		(0)					0		(0)	1	(3)				
**Tumour stage (%)**																							
T1	0		(0)	-				0		(0)	-				6		(18)	2	(6)	-			
T2	0		(0)	-				6		(20)	-				12		(36)	10	(31)	-			
T3	0		(0)	-				4		(13)	-				15		(46)	19	(60)	-			
T4	10		(100)	-				20		(67)	-				0		(0)	1	(3)	-			
**Nodal disease (%)**	5		(50)	-				20		(67)	-				23		(69)	28	(88)	-			
**Metastatic disease (%)**	6		(60)	-				11		(37)	-				1		(3)	1	(3)	-			

*P*-values < 0.05 are highlighted in bold. HOP: head of pancreas; PDAC: pancreatic ductal adenocarcinoma; CA 19 − 9: Carbohydrate antigen 19 − 9; CRP: C-Reactive Protein

### Nanoparticle tracking analysis, electron microscopy and western blotting demonstrate that sec is able to isolate plasma extracellular vesicles

**Fig. 2:**
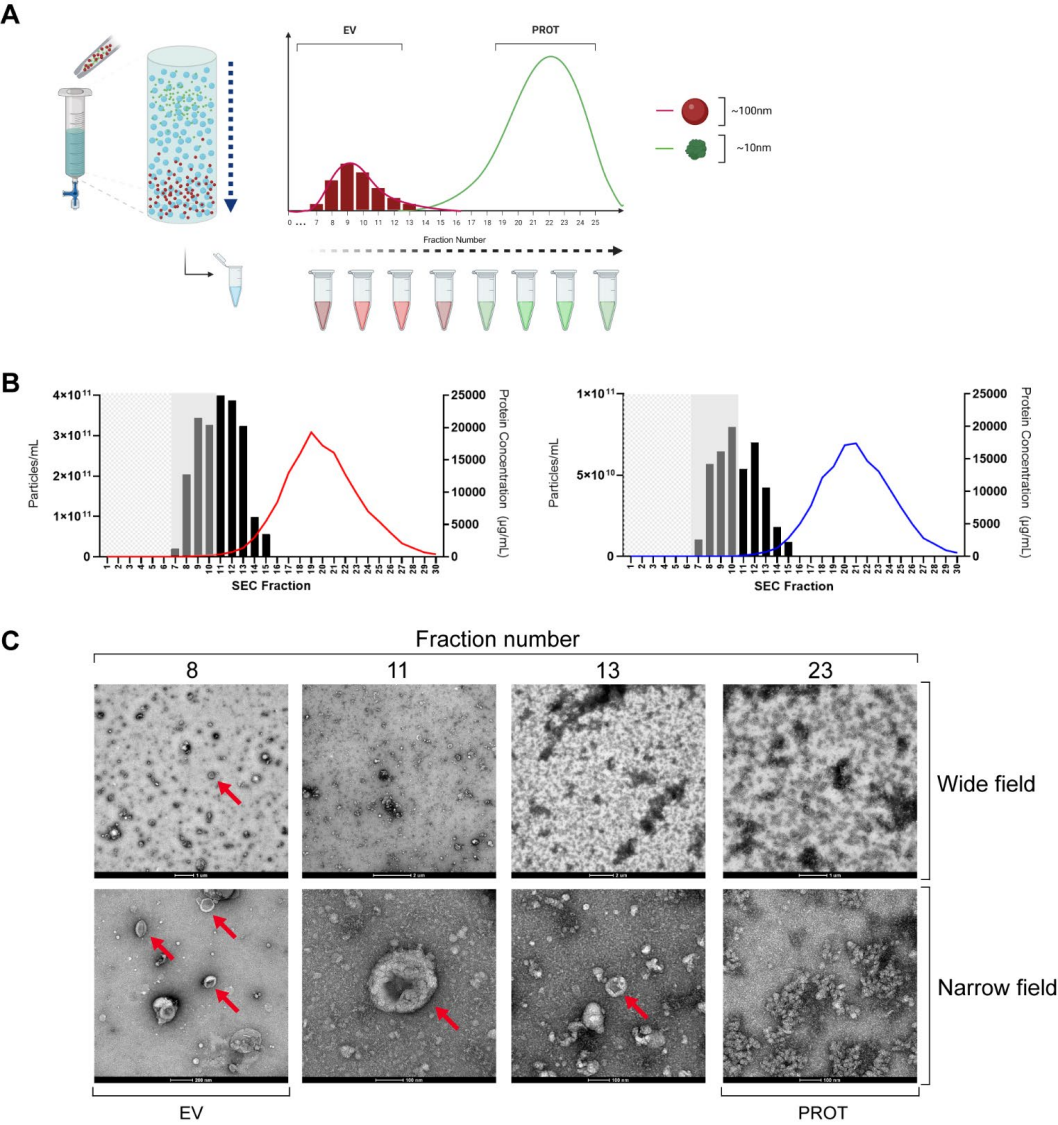
SEC-isolation of plasma EVs. (**A**) SEC using Izon column separates EVs with a size discrimination of 70–100 nm and was performed for a characterisation of 3 PDAC and 3 benign samples. A volume of 1 mL of plasma was loaded to the column and PBS continuously added with 500 µL fractions eluted for subsequent analysis. The column void volume was approximately 3 ml and protein concentrations were measured using a BCA assay. (**B**) Particle and protein concentrations were obtained by NTA and BCA for each of the fractions shown and averages for each fraction are shown in (*left*) PDAC and (*right*) benign disease. Mean particle concentrations are shown as columns with protein levels shown as a coloured trendline (red – PDAC, blue – Benign). (**C**) TEM of a representative PDAC sample shows EVs (red arrows). Images taken at high magnification (60,000–72,000x) are labelled as ‘Narrow field’ whilst intermediate magnification (4-5000x) are labelled as ‘Wide field’. BCA: bicinchoninic acid; EV: extracellular vesicle; NTA: nanoparticle tracking analysis; PBS: phosphate buffered saline; PDAC: pancreatic ductal adenocarcinoma; SEC: size exclusion chromatography; TEM: transmission electron microscopy

SEC makes use of a stationary phase (a gel) which allows a liquid mobile phase (sterile filtered PBS) to pass through. Plasma samples containing complex molecules such as proteins, lipoproteins and EVs are separated and leave the column at a rate proportional to their hydrodynamic volume. With complex mixtures made up of different sized particles, larger molecules are excluded from the gel and are recovered quickly, whilst smaller particles are impeded and elute much later (Fig. 2A).

During characterisation, 1 mL of each plasma sample (3 PDAC, 3 benign) was loaded to the column and eluted with PBS, which was collected in sequential 500 µL fractions. NTA and bicinchoninic acid assays (BCA) were performed, giving average particle and protein concentrations for each fraction, as shown in Fig. 2B. This figure demonstrates a peak in measured particle concentration at fraction 10–11, which corresponds with the expected EV fractions, in both PDAC and benign samples. Furthermore, maximum measured protein concentrations were 19 ± 2.8 mg/mL in PDAC (Fig. 2B, ***left***), and 17 ± 0.3 mg/mL in benign disease (Fig. 2B, ***right***). Transmission electron microscopy (TEM) images of several fractions of a PDAC sample are shown in Fig. 2C. Red arrows highlight a significant number of EVs (particularly in fraction 8) with the expected cup-shaped morphology that occurs when EV preparations are fixed and dried. As the fraction number increases, so does the presence of non-vesicular protein until EVs are poorly resolved at Fraction 23. Additionally, TEM images of a benign plasma sample are included in Supplementary Fig. 1.

**Fig. 3:**
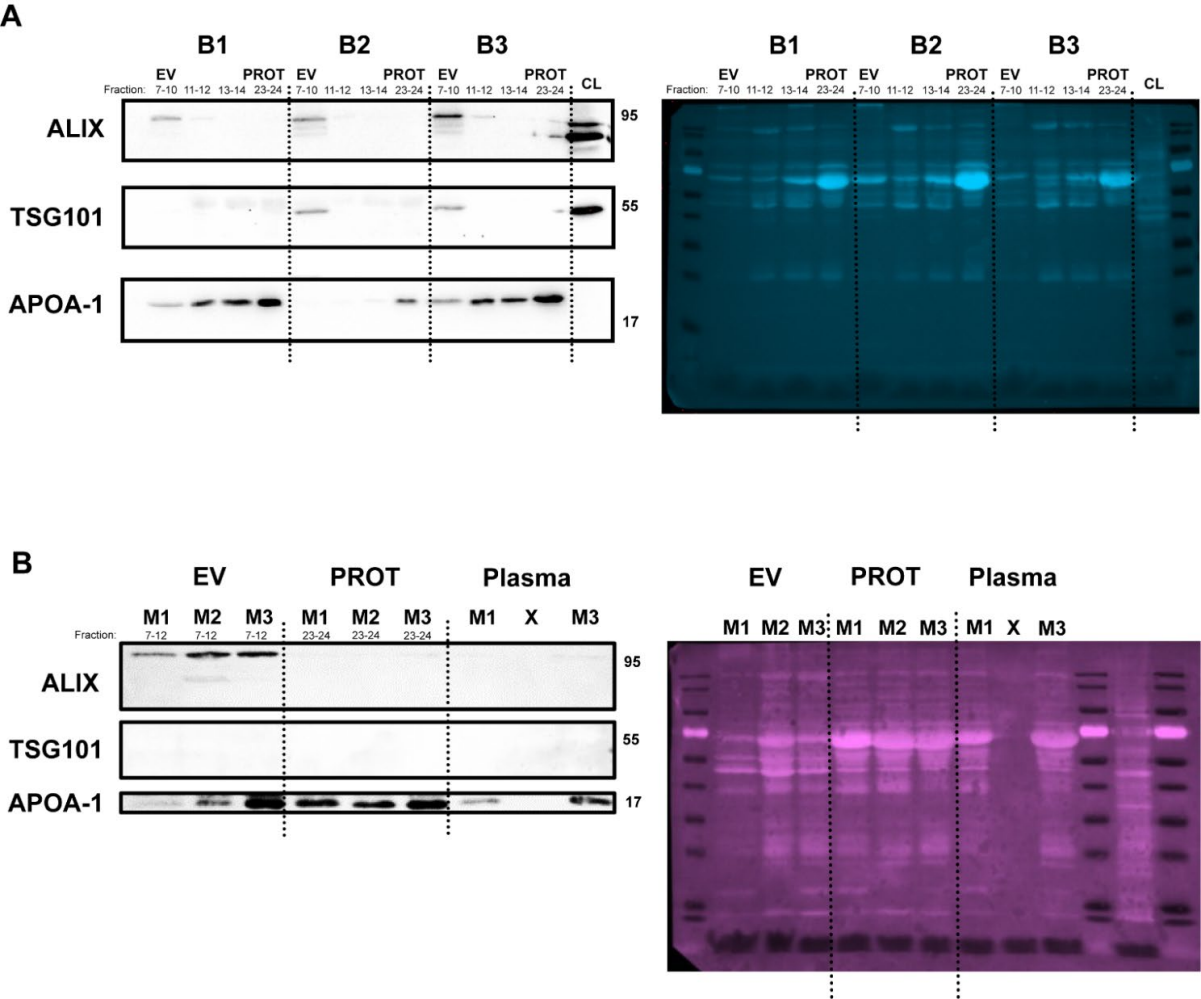
Western blots of specific markers for evaluation of plasma-derived EV preparations. Well-known EV-specific markers, TSG-101 and ALIX, as well as lipoprotein marker APOA-1, were used to assess the purity of the EV preparations. Displayed are (**A**) 3 benign samples (B1, B2, B3), and (**B**) 3 malignant samples (M1, M2, M3). Total protein was stained for normalisation. Variable expression is shown, which is consistent with previously published findings from clinically-obtained samples. EV: extracellular vesicle; PROT: protein

Western blot analyses were performed for 6 samples (3 benign and 3 malignant) using antibodies against EV markers ALIX and TSG101, as well as lipoprotein marker APOA-1 (Fig. 3) in accordance with the MISEV2018, and MISEV2023 update [28, 29]. ALIX and TSG101 are cytosolic proteins which are associated with endosomal sorting complexes required for transport (ESCRT) machinery. ESCRT machinery is involved in the transport of endosomes to the membrane for EV release. In the benign samples (Fig. 3A), ALIX and TSG101 were present in pooled fractions 7–10, and absent from fraction 11 onwards. Conversely, lipoprotein marker APOA-1 appeared throughout the fractions. Although the hypothesis was a complete absence of these protein markers in fractions 7–10, this was found not to be the case. Therefore, although they can be considered as vesicular preparations, they may contain small amounts of additional plasma constituents. In the malignant samples (Fig. 3B), EVs from the malignant samples showed differential (and heterogenous) protein expression consistent with previous studies by both mass spectroscopy [30], and flow cytometry [31]. Results are consistent regarding ALIX expression in PDAC EVs, as well as a relative absence of APOA-1. TSG101 was not detected in these PDAC EVs. Altogether, these experiments show that SEC is able to isolate plasma EVs and provide sufficient data for the assessment of small RNA biomarkers.

### EV characterisation showed that EVs are larger and more numerous in PDAC samples

A total of 61 samples (*n* = 23 benign, 8 CP and 30 PDAC) were analysed by NTA to evaluate EV-characteristics, including median particles size and particle concentration. Supplementary Fig. 2A, shows an example of particle size distribution in a representative (***left***) PDAC and (*right*) benign sample. Average particle size (measured as median) was 156.5 nm (± 13.8) across all samples. While PDAC samples showed a mean particle size of 163.0 nm (± 11.2), benign samples showed a smaller mean particle size of 150.3 nm (± 13.4; Supplementary Fig. 2B, *left*). The EV concentrations followed a skewed distribution, thus median values were calculated. The median EV concentration across all samples was 4.2 × 10^10^ particles/mL (range: 3.5 × 10^9^ – 1.6 × 10^12^), which gives an estimated average yield of 8.4 × 10^10^ EVs/mL of plasma. For PDAC samples, the median EV concentration was 7.7 × 10^10^ particles/mL (range: 3.5 × 10^9^ – 5.2 × 10^11^) whilst in benign disease, the median EV concentration was lower at 2.4 × 10^10^ particles/mL (range: 4.9 × 10^9^ – 1.6 × 10^12^; Supplementary Fig. 2B, *right*). When comparing PDAC and benign samples, there was a significant difference in EV concentrations (*p* = 0.0013) and particle size (*p* = 0.0002). Although this was a statistically significant difference, the biological significance of this difference is unclear due to the spread and overlap, as discussed previously in the literature [32]. To determine the presence of RNAs suitable for sequencing after extraction from pooled EV fractions, RNA was quantified by automated electrophoresis using an Agilent Bioanalyzer. Plots show a predominance of small RNAs < 200 nucleotides with concentrations ~ 100 pg/µL (Supplementary Fig. 2C).

### Small RNA sequencing of plasma-derived EVs reveals differentially expressed MiRNAs and a PDAC associated signature

**Fig. 4:**
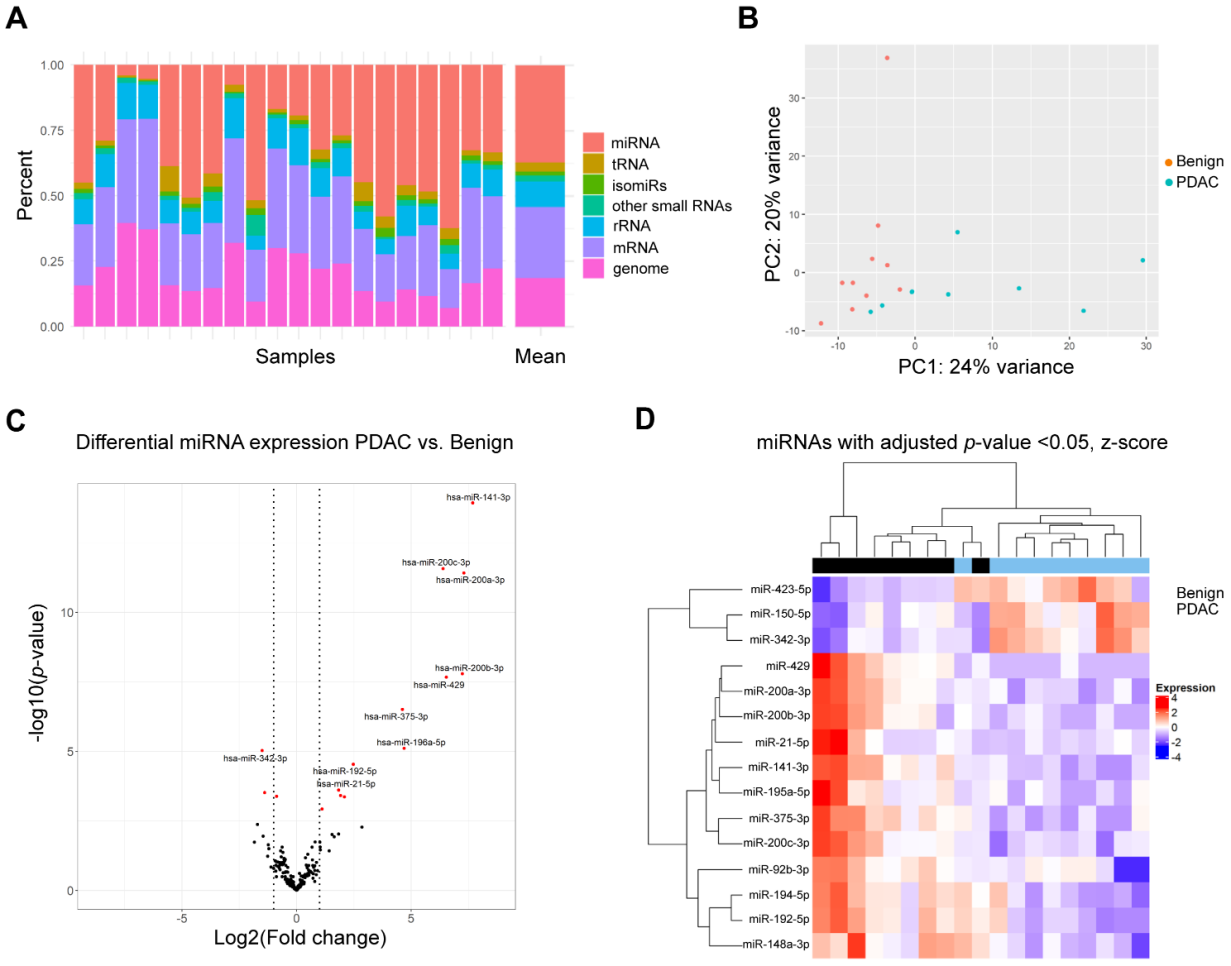
Small RNA sequencing of plasma-derived EVs. (**A**) Relative distribution according to small RNA subtype of mapped reads for each sample shown as stacked barplots. (**B**) Principal component analysis of samples shows clustering of PDAC and benign samples. (**C**) Volcano plot with 15 differentially expressed miRNAs. Red indicates FDR < 0.05. Vertical lines indicate log2 fold change = ± 1. The 10 most significant miRNAs are labelled as shown. (**D**) A heatmap shows significant miRNAs (12 upregulated and 3 downregulated) in the pairwise comparison PDAC vs. benign disease, with relative expression calculated and shown. Red indicates upregulated in the sample, while blue indicates downregulated in the sample. EV: extracellular vesicle; mRNA: messenger RNA; miRNA: microRNA; PDAC: pancreatic ductal adenocarcinoma; FDR: false discovery rate; rRNA: ribosomal RNA; tRNA: transfer RNA

To discover differentially expressed miRNAs, small RNA sequencing was performed for 10 benign and 10 PDAC samples. An average of 13,004,616 total reads (range: 8,402,014–22,524,635) were found and available for differential expression analysis. Reads were mapped and analysis revealed a high variability in mappable small RNAs (Fig. 4A): 37.2% was made up by miRNAs, followed by 27.2% messenger RNAs (mRNA), 18.6% genomic RNAs, 9.6% ribosomal RNAs (rRNA), and 3.4% transfer RNAs (tRNA). Principal component analysis was used to demonstrate variability between data points (Fig. 4B). Two PDAC samples showed low miRNA composition and were hence excluded from further analysis. To reduce noise, miRNAs were filtered by expression (i.e. minimally expressed in 50% of the samples). The top 10 miRNAs with highest expression in plasma EVs across all samples included members of the let-7 family and oncogenic miRNAs, such as miR-21 (see Table 2) [33].

**Table 2:** Top 10 miRNAs with highest expression in EVs from benign and malignant plasma samples

MicroRNA	Mean expression*		
let-7f-5p	11,288.	71	
let-7a-5p	10,855.	55	
miR-122-5p	10,578.	72	
miR-16-5p	9620.	49	
let-7b-5p	8241.	38	
miR-126-3p	5779.	16	
miR-142-3p	4750.	89	
let-7i-5p	3563.	73	
miR-486-5p	2625.	67	
miR-21-5p	2359.	42	

MiRNAs identified by small RNA sequencing were sorted by descending mean expression, which was determined by the expression averaged across all samples and normalized by DESeq2. *Expressed in reads per million

Global profiling for miRNAs was undertaken with cut-offs FDR < 0.05 and log2 fold change > 1. Overall, 12 upregulated and 3 downregulated miRNAs were found to be statistically differentially expressed between PDAC vs. benign disease (Fig. 4C). A heatmap of these miRNAs shows clustering of the PDAC samples (Fig. 4D). The 12 upregulated miRNAs in PDAC (Table 3) included the miR-200 family, consisting of miR-141-3p, miR-200a-3p, miR-200b-3p, miR-200c-3p and miR-429. Upregulated miRNAs are deemed detectable in cancer samples and may be more suitable for diagnostics. MiRNAs were selected for further evaluation based on log2foldchange > 5. Therefore, the 5 upregulated miRNAs of the miR-200 family were taken forward as potential candidates to the validation stage for RT-qPCR.

**Table 3:** MiRNAs that were significantly upregulated in plasma EVs

MicroRNA	Expression*	Log2FoldChange	Standard Error	Adjusted *p*-value
miR-141-3p	197.09	7.6890	0.9961	2.22E-12
miR-200a-3p	251.64	7.3058	1.0524	2.44E-10
miR-200b-3p	88.782	7.2339	1.2811	7.77E-07
miR-429	13.077	6.5386	1.1678	8.18E-07
miR-200c-3p	235.39	6.3926	0.9142	2.44E-10
miR-196a-5p	13.382	4.6913	1.0493	0.0002
miR-375-3p	13.756	4.6179	0.9026	0.0000
miR-192-5p	305.94	2.4751	0.5925	0.0006
miR-194-5p	111.04	2.0845	0.5925	0.0059
miR-92b-3p	16.172	1.9158	0.5403	0.0059
miR-21-5p	2359.4	1.8383	0.5021	0.0048
miR-148a-3p	415.54	1.1132	0.3436	0.0151

Shown are significant (adjusted p-values < 0.05) miRNA candidates identified by small RNA sequencing and sorted by descending log fold change values. Expression averaged across all samples with normalization by DESeq2 to allow direct comparison between the samples. Standard errors are of the log2 fold change and p-values are adjusted using the Benjamini-Hochberg method. In bold are candidates used in later validation. *Expressed in reads per million

### Small RNA sequencing of plasma-derived circulating miRNAs fails to show significant differential expression

As part of a discovery study into diagnostic miRNAs to discriminate PDAC from benign patients, both plasma EV-derived miRNAs and plasma cell-free miRNAs (cf-miRNA) were assessed (Supplementary Fig. 3). Direct small RNA sequencing of miRNAs from the total plasma was performed in 41 samples for the pairwise comparison of malignant vs. benign disease. After controlling for multiple comparisons, two cf-miRNA candidates (miR-144-5p and miR-29c-3p) showed significant differential expression (Supplementary Fig. 4A). ROC curves for miR-144-5p and miR-29c-3p are demonstrated in Supplementary Fig. 4B and revealed AUCs of 0.828 and 0.808, respectively. Given the low number of candidates found, it was decided not to proceed to validation with these candidates and focus on EV-miRNAs as our primary end-point.

### Technical validation of EV-miR-200 family shows validity of differential expression

**Fig. 5:**
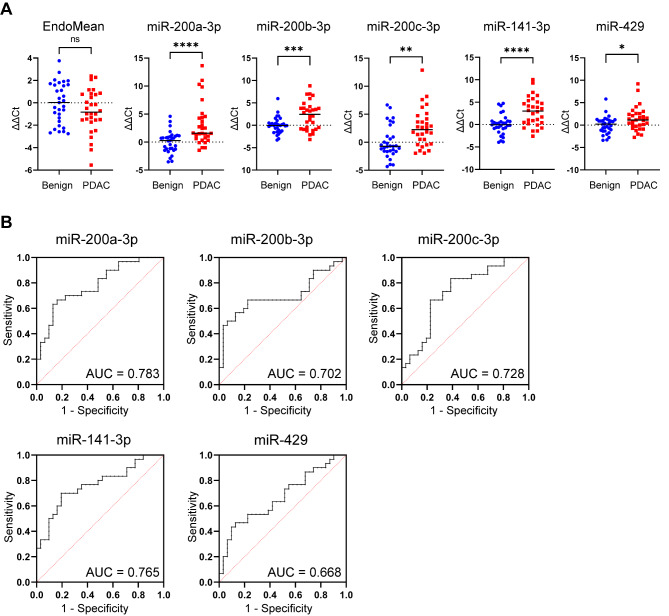
Validation of miR-200 family in plasma EV samples using RT-qPCR. (**A**) Expression of the EndoMean (the geometric mean of endogenous normalisers miR-23a, miR-26a and exogenous UniSp6) and the miR-200 family (miR-200a, miR-200b, miR-200c, miR-141 and miR-429) in plasma EV samples for the pairwise comparison: 30 PDAC vs. 31 benign. (**B**) Receiver operating curves with area under the curve (AUC) for each individual miR-200 family member is generated from logistic regression of the RT-qPCR expression. *p < 0.05, **p < 0.005, ***p < 0.0005, ****p < 0.0001

Following RNA isolation of 61 plasma EV samples (23 benign, 8 chronic pancreatitis (CP) and 30 PDAC), RT-qPCR was performed for the miR-200 family. In order to normalise RT-qPCR data, stable endogenous miRNAs were identified by applying NormFinder algorithms to the RNA-sequencing data. The geometric mean of stably expressed miR-23a, miR-26a and exogenous UniSp6 was defined as the EndoMean. Average Ct values were calculated for technical triplicate RT-qPCR assays of candidate miRNAs. Relative expression after normalisation gave rise to differential expressions analysis which confirmed upregulation in PDAC for each of the miR-200 family members, as shown in Fig. 5A. Logistic regression was performed to assess the diagnostic value of each miRNA individually, generating ROC curves and corresponding AUCs (Fig. 5B). The individual AUC for miR-200a was 0.783 (95%CI 0.668–0.897), for miR-200b 0.702 (95%CI 0.564–0.840), for miR-200c 0.728 (95%CI 0.600-0.856), for miR-141 0.765 (95%CI 0.644–0.885) and miR-429 0.668 (95%CI 0.531–0.804). In addition, RT-qPCR data of the EV-miR-200 family expression in plasma from healthy donors (*n* = 14) showed Ct values > 40, indicating very low expression, or absence of these miRNAs (Supplementary Table 4). These values were above the Ct value cut-off of 40. Therefore, we concentrated on distinguishing benign disease from malignant disease.

**Fig. 6:**
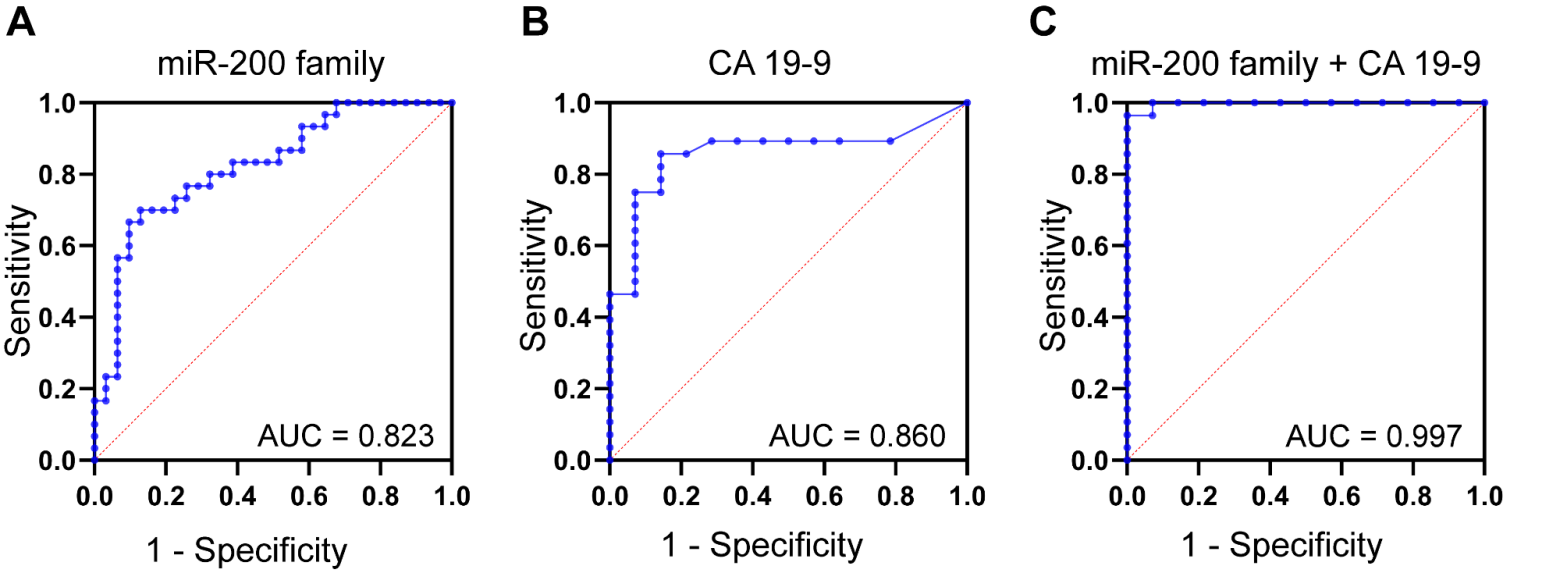
Diagnostic value of the miR-200 family signature, which improved by addition of CA 19 − 9. Receiver operating characteristic curves and corresponding area under the curve values (AUCs) for (**A**) the miR-200 family (PDAC *n* = 30 vs. benign disease *n* = 31), (**B**) CA 19 − 9 (PDAC *n* = 28 vs. benign disease *n* = 14) and (**C**) the combination of the miR-200 family and CA 19 − 9 (PDAC *n* = 28 vs. benign disease *n* = 14). Patients with missing values for CA19-9 were not included in the analyses of (**B**) and (**C**). *p < 0.05, **p < 0.005, ***p < 0.0005, ****p < 0.0001

In combination, EV-miR-200 family showed an improved diagnostic accuracy with an AUC of 0.823 (95% CI 0.717–0.928; Fig. 6A). The calculated AUC for CA 19 − 9 in this cohort was 0.860 (95% CI 0.741 to 0.979; Fig. 6B), which was limited by missing values particularly in the benign population (17 out of 31; 54%). The addition of CA 19 − 9 to the model generated an AUC of 0.997 (95% CI 0.989-1.000; Fig. 6C).

### Clinical validation shows EV-miR-200 family is also upregulated in an independent cohort of patients

**Fig. 7:**
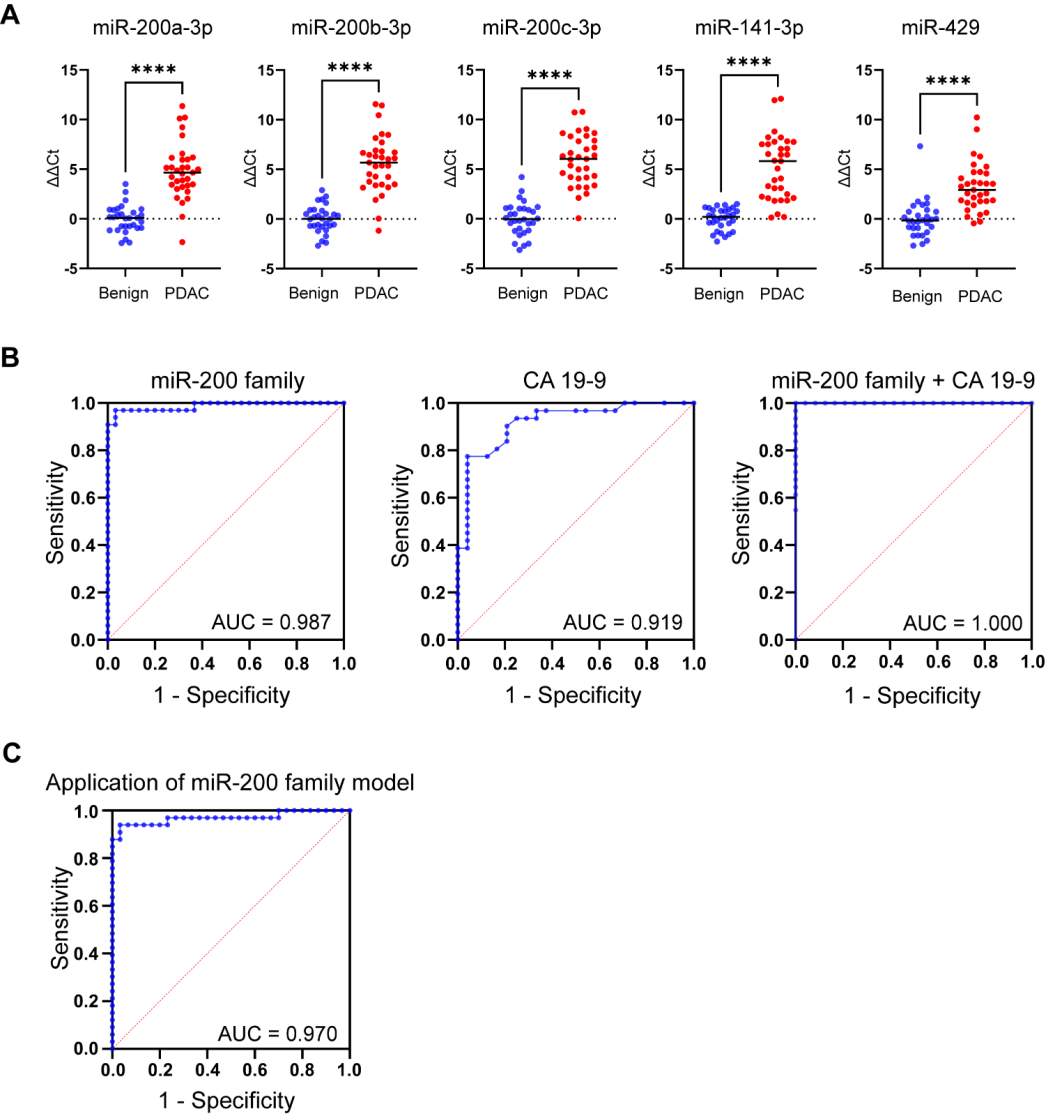
Confirmation of differential expression and accuracy of the EV-miR-200 family model in an independent cohort. (**A**) RT-qPCR results of EV-miR-200 family members in the clinical validation cohort for the pairwise comparison: PDAC (*n* = 33) vs. benign disease (*n* = 30). (**B**) ROC curves and corresponding AUCs for (*left)* the EV-miR-200 family signature (miR-200a-3p, miR-200b-3p, miR200c-3p, miR-141-3p and miR-429), (*middle*) CA 19 − 9, and (*right*) the combination of CA 19 − 9 and the EV-miR-200 family signature, for predicting PDAC. (**C**) Diagnostic accuracy of the EV-miR-200 family model for predicting PDAC (vs. benign) when applied to the clinical validation cohort, which consisted of benign (*n* = 30) and PDAC (*n* = 33) samples. AUC: area under the curve values; PDAC: pancreatic ductal adenocarcinoma; ROC: receiver operating characteristic. *p < 0.05, **p < 0.005, ***p < 0.0005, ****p < 0.0001

Differential expression of the EV-miR-200 family (miR-200a-3p, miR-200b-3p, miR200c-3p, miR-141-3p and miR-429) was confirmed in an independent validation cohort of 30 benign and 33 PDAC samples (Fig. 7A). Logistic regression analysis generated an AUC of 0.987 (95%CI 0.964-1.000; Fig. 7B, ***left***) for diagnosing PDAC. When comparing CA19-9 measurements in 31 PDAC and 24 benign samples, CA19-9 demonstrated an AUC of 0.919 (95%CI 0.846–0.993) for predicting PDAC (Fig. 7B, ***middle***). When combined with the EV-miR-200 family, it improved to an AUC of 1.00 (95%CI 1.00–1.00; Fig. 7B, ***right***). Similar results were found when comparing benign disease with both PDAC and CCA, as illustrated in Supplementary Fig. 5, indicating that these miRNAs have potential to discriminate benign disease from both PDAC and CCA.

### A novel plasma EV-miRNA Model for diagnosing PDAC in the clinical validation cohort

A plasma EV-miRNA dichotomous outcome (PDAC or benign) model was generated by logistic regression analysis using ∆Ct RT-qPCR miRNA expression data of the technical validation cohort. CA 19 − 9 was omitted from the models, as 54% of the patients with benign disease in the technical validation cohort did not have a recorded CA 19 − 9 level, and not all patients with malignant disease secrete CA 19 − 9. The miRNA model was then tested in the independent clinical validation cohort. When comparing PDAC (*n* = 30) and benign disease (*n* = 31) in the technical validation cohort, the EV-miR-200 family generated a diagnostic model (*y* = 3.559 + miR-200a-3p*0.3444 + miR-200a-3p*0.0757 + miR-200c-3p*0.0722 + miR-141*0.3049 + miR-429*-0.4012) with the most optimal cut-off at 0.5209. Application of this EV-miR-200 family model to the independent validation cohort showed a sensitivity of 100.0%, specificity of 88.2%, NPV of 100.0% and PPV of 87.9%, with an AUC of 0.970 (95%CI 0.925-1.000; *p* < 0.0001; Fig. 7C). When assessed in early-stage PDAC only (stage I/II, *n* = 25) vs. benign disease (*n* = 30), the model generated an AUC of 0.960 (95%CI 0.902-1.00; *p* < 0.0001; Supplementary Fig. 5A). In addition, in the subset of patients (*n* = 12) with T1 (i.e. tumour ≤ 2 cm in greatest dimension, 8th edition of the AJCC), or T2 (i.e. tumour > 2 cm in greatest dimension, but less than ≤ 4 cm), the model showed an AUC of 0.936 (95%CI 0.825-1.00; *p* < 0.0001); Supplementary Fig. 6B). For the comparison benign disease vs. PDAC plus CCA, this model showed a sensitivity of 100.0%, specificity of 83.3%, NPV of 100.0% and PPV of 90.8%, with an AUC of 0.984 (95%CI 0.961-1.000; *p* < 0.0001; Supplementary Fig. 5C).

## Discussion

This study aimed to find a diagnostic miRNA profile in SEC isolated plasma EVs. Differential expression analysis of small RNA sequencing data showed significant upregulation of the miR-200 family in PDAC vs. benign pancreaticobiliary disease, which was validated using RT-qPCR in a cohort of 30 PDAC vs. 31 benign samples. Upon combining the EV-miR-200 family members (miR-200a, miR-200b, miR-200c, miR-141, and miR-429), the diagnostic miRNA signature revealed an AUC of 0.823 (95% CI 0.717–0.928). Adding of CA19-9 improved the diagnostic accuracy to 0.997 (95% CI 0.989-1.000), but this was biased by data collection limitations. Expression levels of EV-miR-200 family members were also assessed in an independent cohort of patients, including both PDAC and CCA, and were found upregulated in both malignancies. Testing the EV-miR-200 family model a priori in the independent clinical validation cohort (32 PDAC vs. 30 benign) predicted PDAC with an impressive sensitivity of 100.0%, specificity of 88.2%, NPV of 100.0%, PPV of 88.7%, and an AUC of 0.970 (95% CI 0.925-1.000; *p* < 0.0001). In a broader comparison involving both CCA and PDAC (32 CCA vs. 33 PDAC) against benign disease (30 cases), the model also exhibited a good predictive performance with sensitivity, specificity, NPV, PPV, and AUC values of 100.0%, 83.3%, 100.0%, 90.8%, and 0.984 (95% CI 0.961-1.000; *p* < 0.0001), respectively, for identifying malignant pancreaticobiliary disease.

The EV-miR-200 family has been previously identified in blood of patients with PDAC. Reese and colleagues conducted a study where ultracentrifugation was employed to isolate serum EVs, revealing elevated levels of miR-200b and miR-200c in PDAC patients (*n* = 56), as compared to both healthy controls (*n* = 22) and patients with chronic pancreatitis (*n* = 11), determined by RT-qPCR [34]. MiR-200b was able to distinguish PDAC from healthy patients with an AUC of 0.79, and an AUC of 0.77 in distinguishing from chronic pancreatitis. Similarly, our study showed a diagnostic AUC of 0.70 for miR-200b in identifying PDAC from benign controls. However, we chose to utilise multiple miRNAs for our diagnostic model, which significantly improved diagnostic accuracy during discovery and validation (EV-miR-200 model AUC = 0.97 in benign vs. PDAC; Fig. 7C). Another study by Nakamura et al. assessed plasma EV-miRNAs in 44 patients with PDAC and 57 non-disease controls and identified 17 EV-miRNAs from RNA-seq that were significantly upregulated in PDAC [35]. Their final validated model consisted of 5 cell-free (AUC = 0.84) and 8 EV-miRNAs (AUC = 0.89), of which 3 were the miR-200 family members that we also examined. In contrast with non-disease controls, our study acquired samples from benign pancreaticobiliary disease, such as chronic pancreatitis and cholelithiasis, and we applied the diagnostic model to an independent cohort to test its accuracy.

The miR-200 family is an important modulator of epithelial-to-mesenchymal transition (EMT) [36–38], , which is one of the imperative mechanisms in cancer metastasis in many tumour types, as exemplified in breast cancer and insulinoma [39–41]. Activation of the EMT leads to stimulation of several well-recognised transcription factors (i.e. SNAI1, SLUG, TWIST, *Zeb1/2*) that contribute to dedifferentiation, loss of apicobasal polarity, loss of cell-cell adhesion and increased motility, acquiring instead a mesenchymal phenotype [42]. During TGF-ß-mediated EMT, there is a significant decrease in miR-200 family expression, which in turn leads to the inhibition of E-cadherin through acting on Zinc Finger E-box binding homeobox 1 (*Zeb1*) and *Zeb2* [36, 38, 43]. Indeed, enforced in vitro expression of the miR-200 family prevented EMT, and induced mesenchymal-to-epithelial (MET) transition. In vivo, ablation of the *Zeb1* allele in the PDAC KPC mouse models showed decreased tumour progression and metastasis, while re-expression of *Zeb1* led to progression and invasive stages [37, 44]. Therefore, we postulate that the miR-200 family may be secreted into EVs by PDAC cells to move these molecules away from the tumour to allow further EMT, and to promote distant pre-metastatic niche formation through MET.

Cancers are unlikely to be present mutually exclusive as epithelial or mesenchymal, but may instead exist in a continuum of partial EMT states [45]. Even in PDAC cell lines, there is a range of phenotypes with more mesenchymal cell lines, such as Mia-PaCa-2, showing lower miR-200 expression compared to epithelial-like BxPC-3 (with higher miR-200 expression) [46]. This “EMT-MET plasticity” has been found to be a key feature in PDAC metastatic organotropism, influencing whether cancer cells metastasise to liver or lung. In murine models, cancer cells that maintain a stable mesenchymal state tend to develop undifferentiated tumours but fewer liver metastases in murine models [47]. It is therefore possible that miR-200 tissue expression can lead to both an epithelial phenotype and, paradoxically, increased metastatic disease. Additionally, miR-200 (in particular miR-200c) has been shown overexpressed in pancreatic islets in diabetic mice and lead to ß-cell apoptosis through a *Zeb1* independent pathway and possibly through Ypel2, a potential oncogene, leading to type 2 diabetes [48]. The elevated levels of miR-200c may, therefore, be one mechanism contributing to impaired glucose metabolism and new onset diabetes in patients with PDAC [49].

This study was limited by the inclusion of patients with predominately late-stage disease in the initial discovery and technical validation cohorts, as these patients were recruited at first presentation with jaundice, rather than those only selected for upfront surgery. However, in the independent validation cohort, 54% of the patients with PDAC were early stage (T1-T2) and 76% were stage I/II, which indicates that our model is equally applicable as a marker of early-stage malignant disease. Furthermore, SEC was utilised as method for isolation, which has limitations including the co-precipitation of lipoproteins. These lipoproteins may also contain RNA, and might have been co-isolated with the vesicles [50]. In order to determine if this was a possibility, fractions 11–14 from the EV isolation were also collected with high expression of APOA-1 (Fig. 3). These fractions were not included in the EV preparations and additional experiments in a small group of PDAC samples (*n* = 6) showed a significant downregulation of the miR-200 family in these lipoprotein fractions. Although ultracentrifugation is the most common used method for isolation, it also isolates non-EVs, requires skill, intense labour and is time-consuming [51]. It is our opinion that its limited scalability is a major roadblock for clinical applications and that studies have shown SEC is rapid, has a lower protein/vesicle ratio (suggesting a higher purity) and shows higher yields, while preserving the EV proteome [52]. This study, totalling 156 patients, used two separate validation cohorts to verify the results of small RNA-sequencing data, which indicated that the EV-miR-200 family may have a role in the diagnosis of PDAC and/or CCA. Further follow-up studies are in progress to assess the prognostic and predictive value of these miRNAs in a larger multicentric cohorts.

## Conclusion

This study used SEC as a novel and rapid way of processing plasma samples for biomarker discovery and validation in PDAC. We have designed a pipeline to isolate and characterise plasma-derived EVs from clinical samples in a treatment-naïve population. The plasma-derived EVs contained miRNAs, notably miR-141-3p, miR-200a-3p, miR-200b-3p, miR-200c-3p and miR-429, that can potentially serve as novel biomarkers in the plasma EVs of patients with PDAC and CCA (although this latter requires further validation). Applied as the diagnostic EV-miR-200 family model to our independent validation cohort, the model showed an outstanding AUC (0.97), and a predicted sensitivity and specificity of 100.0% and 88%, respectively. Together with serum CA 19 − 9, this may provide additional early diagnostic information and should be further validated in larger, multicentric trials.

### Electronic supplementary material

Below is the link to the electronic supplementary material.

Supplementary Material 2

Supplementary Material 4

## Data Availability

The datasets supporting the conclusions of this article are available in the EV-TRACK knowledgebase (EV-TRACK ID: EV210164. Van Deun J, et al. EV-TRACK: transparent reporting and centralizing knowledge in extracellular vesicle research. Nature methods. 2017;14(3):228 − 32).

## Abbreviations

AUC: Area Under the Curve
BCA: Bicinchoninic Acid
BTC: Biliary Tract Cancer
CA 19 − 9: Carbohydrate Antigen 19 − 9
CCA: Cholangiocarcinoma
CEA: Carcinoembryonic Antigen
cf: cell-free
CI: Confidence Interval
CRP: C-Reactive Protein
EDTA: Ethylenediaminetetraacetic
EMT: Epithelial-to-Mesenchymal Transition
ERCP: Endoscopic Retrograde Cholangiopancreatography
EV: Extracellular Vesicle
FDR: False Discovery Rate
FGF: Fibroblast Growth Factor
HITS-CLIP: High-Throughput Sequencing of RNA Isolated by Crosslinking Immunoprecipitation Procedure
HOP: Head of Pancreas
MET: Mesenchymal-to-Epithelial Transition
miR: microRNA
NPV: Negative Predictive Value
NTA: Nanoparticle Tracking Analysis
PDAC: Pancreatic Ductal Adenocarcinoma
PPV: Positive Predictive Value
ROC: Receiver Operating Characteristic
RPM: Reads Per Million Reads
RT-qPCR: Real-Time quantitative Polymerase Chain Reaction
SD: standard deviation
SEC: Size Exclusion Chromatography
TGF-β: Transforming Growth Factorβ
TNM: Tumor, Node and Metastasis
UC: Ultracentrifugation
ZEB: Zinc Finger E-box Binding Homeobox
