# Supplementary material for: Unlocking the diagnostic power of plasma extracellular vesicle miR-200 family in pancreatic ductal adenocarcinoma
Source: J Exp Clin Cancer Res. 2024 Jul 8;43:189. doi: 10.1186/s13046-024-03090-z (PMC11229220; doi:10.1186/s13046-024-03090-z)
Supplement: Supplementary file 1 — Supplementary Material 2 [file 13046_2024_3090_MOESM2_ESM.pdf]

INTERNATIONAL SOCIETY for EXTRACELLULAR VESICLES

MIBlood-EV

Standardized Reporting Tool for Blood EV Research (Human)

STUDY INFORMATION

1.0 Manuscript title	Unlocking the diagnostic power of plasma extracellular vesicle miR-200 family in pancreatic ductal adenocarcinoma		
1.1 Corresponding author (Name and Email)	Adam Frampton (a.frampton@imperial.ac.uk), Elisa Giovannetti (elisa.giovannetti@gmail.com)		
1.2 Institution name	Imperial College London, Amsterdam UMC Location Vrije Universiteit Amsterdam		
1.3 Time period of experiment (e.g. 2022-2024)	2017-2024	1.4 Number of samples	162
1.5 Cargo of interest	☑ Vesicles ☐ Protein ☑ RNA ☐ DNA ☐ Other:		
1.6 Biospecimen type	☑ Plasma ☐ Serum	1.7 Biospecimen state	Frozen
1.8 Source of frozen specimens	Prepared by the lab	1.9 Years of collection (range)	2017-2023

BLOOD COLLECTION AND PROCESSING

2.0 Patient fasting status	Fasted	2.1 Fasting length (e.g. hours/days)	8 hours
2.2 Anatomical access site	Antecubital fossa	2.3 Needle diameter (e.g. gauge)	16G-23G
2.4 Blood volume collected (mL)	2-16		
2.5 Plasma anticoagulant	☑ EDTA ☐ Citrate ☐ Heparin ☐ Other:		
2.6 Serum tube type		2.7 Serum clotting time (minutes)	
2.8 Time between collection and first centrifugation (range in hours)	4		
2.9 Transport temperature	Room temp (~22°C)	2.10 Transport condition of tubes	Unknown
2.11 Centrifuge brand and model	Centrifuge 5810R, A-4-81 rotor, Eppendorf		
2.12 Bucket rotor type	Swing	2.13 Number of centrifugation cycles	One
2.14a 1st Centrifugation speed (RCF in x g)	2500	2.14b 1st Centrifugation time (minutes)	10
2.15 1st Rotor brake	On	2.16 1st Centrifugation temperature	Room temp (~22°C)
2.17a 2nd Centrifugation speed (RCF in x g)		2.17b 2nd Centrifugation time (minutes)	
2.18 2nd Rotor brake		2.19 2nd Centrifugation temperature	
2.20 Additional processing steps (e.g. filtration)			
2.21 Storage tubes (brand, type, source, catalog number)	1.5 mL Eppendorf safe-lock tubes (Catalog no. 0030123328)		
2.22 Storage temperature	-80°C	2.23 Length of storage (range in years)	0-2

PLASMA/SERUM QUALITY CONTROL

3.0 Number of freeze-thaw cycles (range)	0-1		
3.1 Thawing temperature	on ice	3.2 Thawing duration (minutes)	120

Hemolysis

3.3 Presence of hemolysis	No	3.4 Frequency of hemolyzed samples (e.g. <25%, 25-50%)	0%
3.5 Method used	Spectrophotometry	3.6 RBC count (Median, 95% CI, N)	
3.7 RBC counter brand and type			
3.8 Spectrophotometry hemoglobin concentration (mean g/L)	0		
3.9 Spectrophotometer brand, model and wavelength measured (e.g. 414 nm)	Nanodrop 1000 (Thermo Scientific) spectrophotometer (414nm)		
3.10 Hemolized samples were discarded	Yes		

Platelets

3.11 **Presence of platelets**	Not Tested	3.12 **Method used (e.g. Flow Cytometry)**	
3.13 **Marker(s) used (e.g. CD61, CD41)**			
3.14 **Concentration (median, 95% CI, N)**			
3.15 **Platelet counter instrument brand, type and limit of detection (cells/L)**			
3.16 **Flow cytometer brand and type**			
3.17 **Flow cytometry size and fluorescence ranges of detection in nanometers and MESF, respectively**			

Lipoproteins

3.18 **Presence of lipoproteins**	Yes	3.19 **Method used (WB, ELISA, FC)**	Western-blot
3.20 **Spectrophotometry L-index**			
3.21 **Spectrophotometer brand, model and wavelength measured (e.g. 700 nm)**			
3.22 **WB Marker(s) used (e.g. Apo B)**	ApoA1		
3.23 **Western blot images provided in manuscript?**	Yes		
3.24 **Flow cytometry marker(s) used (e.g. ApoB)**			
3.25 **Flow cytometry concentration (median, 95% CI, N)**			
3.26 **Flow cytometer brand and type**			
3.27 **Flow cytometry size and fluorescence ranges of detection in nanometers and MESF, respectively**			
3.28 **Brand and catalog number for ELISA kit used for each Apolipoprotein test (e.g. ApoB)**			
3.29 **Concentration measured for each Apolipoprotein tested (mean µg/mL)**			
