# Supplementary material for: Unlocking the diagnostic power of plasma extracellular vesicle miR-200 family in pancreatic ductal adenocarcinoma
Source: J Exp Clin Cancer Res. 2024 Jul 8;43:189. doi: 10.1186/s13046-024-03090-z (PMC11229220; doi:10.1186/s13046-024-03090-z)
Supplement: Supplementary file 2 — Supplementary Material 4 [file 13046_2024_3090_MOESM4_ESM.docx]

# Additional File 1

**Unlocking the diagnostic power of plasma extracellular vesicle miR-200 family in pancreatic ductal adenocarcinoma**

Daniel S.K. Liu^1,†^, Jisce R. Puik^2,3,†^, Bhavik Y. Patel^4,5^, Morten T. Venø^6,7^, Mahrou Vahabi^3^, Mireia Mato Prado^1,8^, Jason P. Webber^9^, Eleanor Rees^1^, Flora M. Upton^1^, Kate Bennett^1^, Catherine Blaker^1^, Benoit Immordino^10^, Annalisa Comandatore^11^, Luca Morelli^11^, Shivan Sivakumar^12^, Rutger-Jan Swijnenburg^2,3,13^, Marc G. Besselink^3,13^, Long R. Jiao^1^, Geert Kazemier^2,3^, Elisa Giovannetti^3,14,‡,^**^*^**, Jonathan Krell^1,‡^ & Adam E. Frampton^1,4,5,‡^**^,*^**

**Correspondence:** Adam E. Frampton, HPB Surgical Unit, Royal Surrey County Hospital, Guildford, Surrey, UK. Email: [a.frampton@imperial.ac.uk](mailto:a.frampton@imperial.ac.uk); adam.frampton@surrey.ac.uk. Elisa Giovannetti, Laboratory of Medical Oncology, Amsterdam UMC location VUmc, Amsterdam, NL. Email: [elisa.giovannetti@gmail.com](mailto:elisa.giovannetti@gmail.com).

**
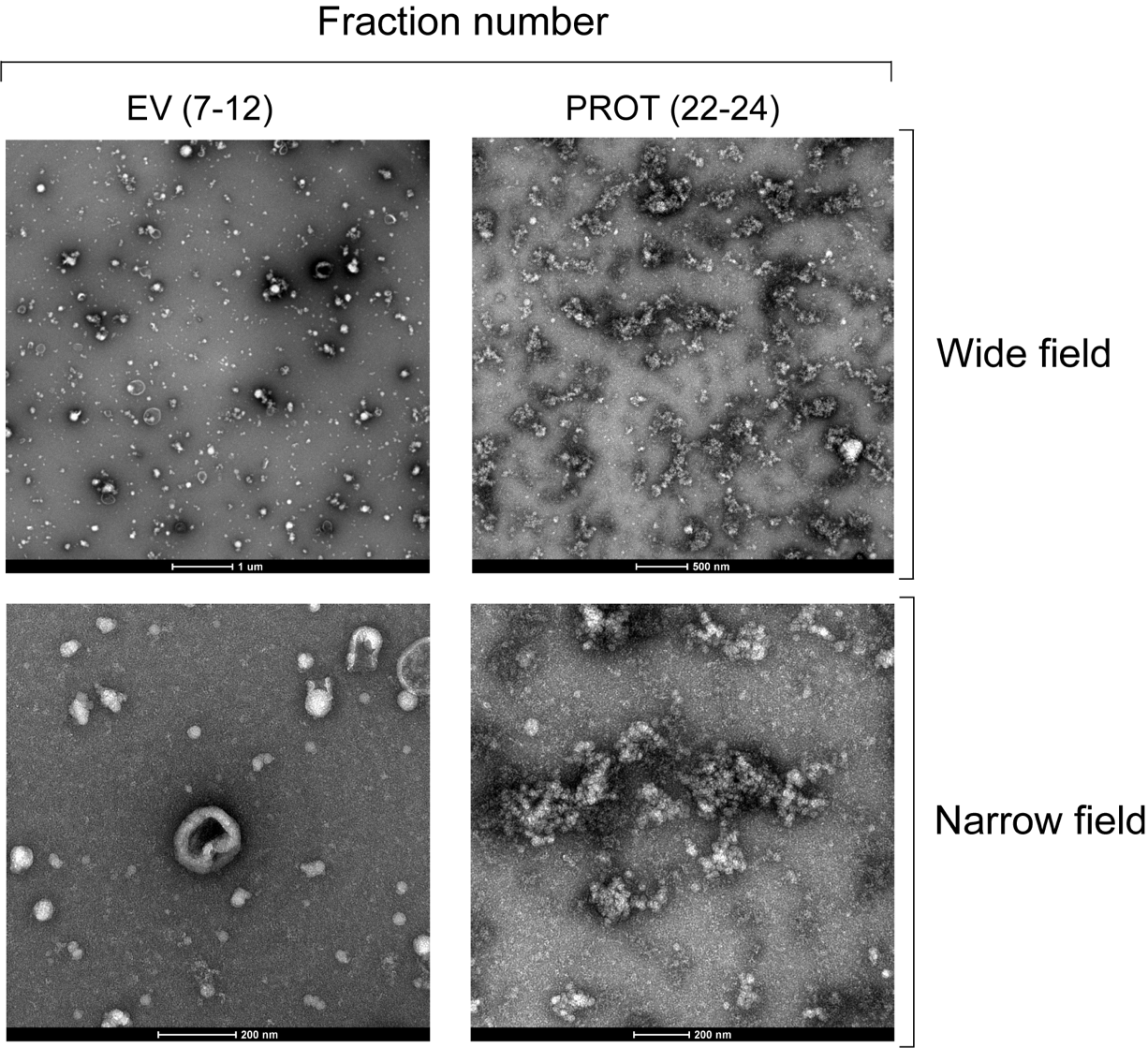
Supplementary Figure 1. Transmission Electron Microscopy images of a benign plasma sample.** Images taken at high magnification (60,000-72,000x) are labelled as ‘Narrow field’ whilst intermediate magnification (4-5000x) are labelled as ‘Wide field’.
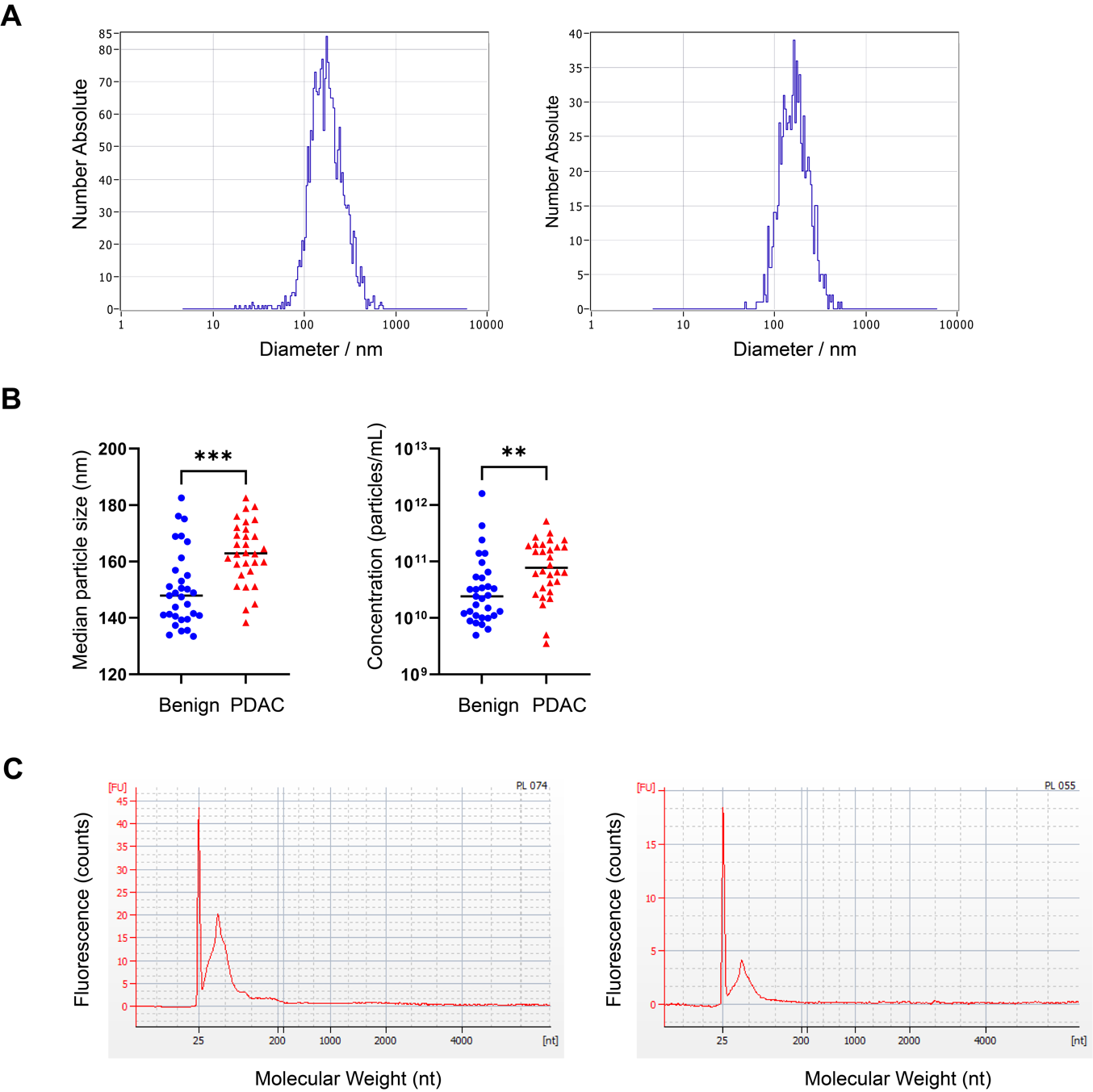


**Supplementary Figure 2. Characteristics of plasma EVs.** (**A**) An example of particle size distribution in a representative (*left*) PDAC and (*right*) benign sample determined by nanoparticle tracking analysis. (**B**) (*Left*) EV median particle size (nm) and (*right*) concentration (particles/mL) for the pairwise comparison benign disease (n=31) vs. PDAC (n=30). (**C**) Automated electrophoresis results demonstrate the presence of small RNAs (<200 nucleotides) in a representative (*left*) PDAC and (*right*) benign sample. **p*<0.05, ***p*<0.005, ****p*<0.0005. EV: extracellular vesicle; PDAC: pancreatic ductal adenocarcinoma.

**Supplementary Figure 3. Schematic overview of cell-free and EV-derived miRNA evaluation in PDAC and benign plasma samples.** EV: extracellular vesicle; PDAC: pancreatic ductal adenocarcinoma.


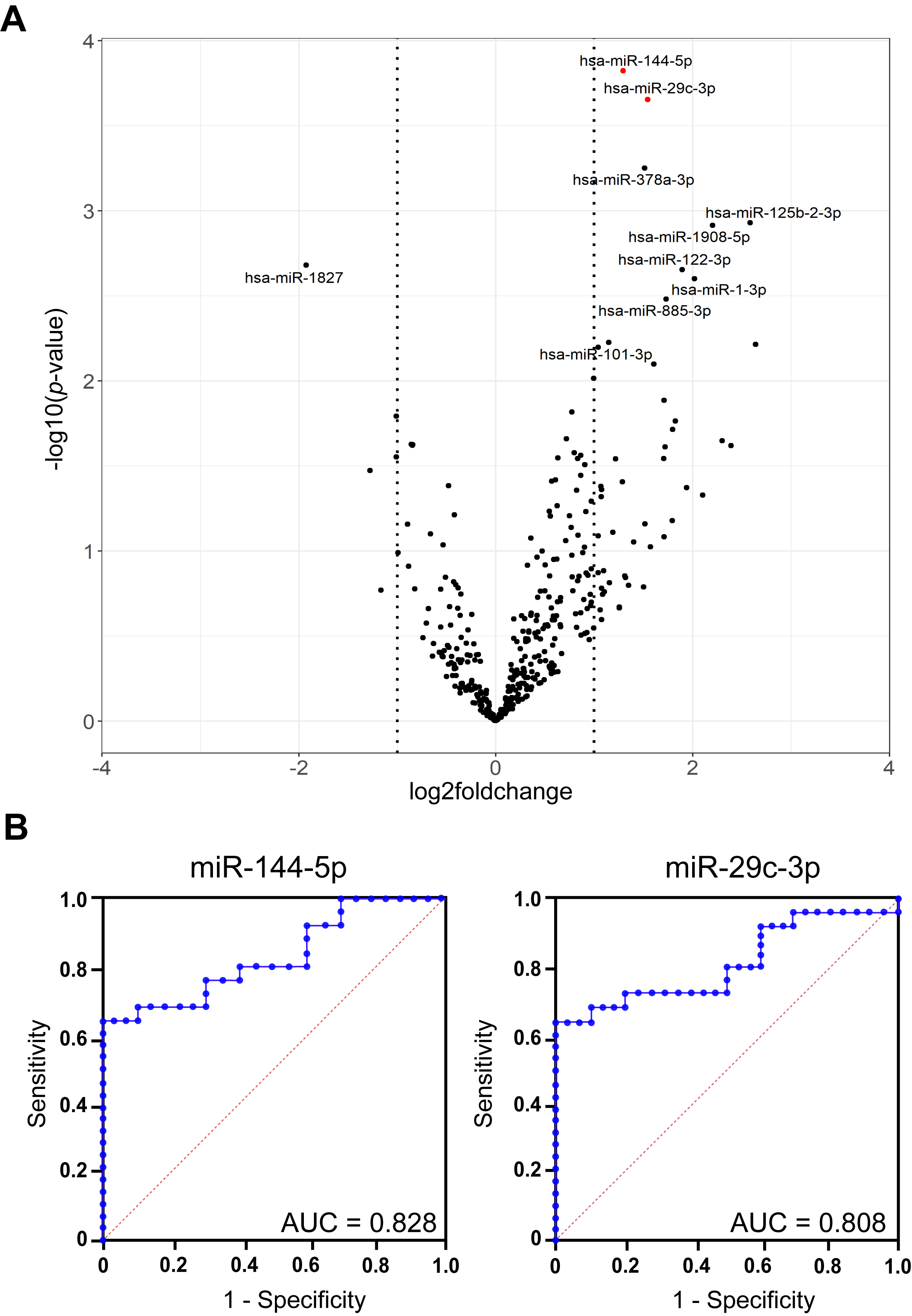


**Supplementary Figure 4. Differential expression analysis of plasma cell-free miRNAs for the pairwise comparison PDAC vs. benign disease.** (**A**) Volcano plot with differentially expressed miRNAs. Red indicates FDR <0.05. Vertical dashed lines indicate log2 fold change = ±1. The 10 most significant miRNAs are labelled as shown. (**B**) ROC curves and corresponding AUCs of (*left*) cell-free miR-144-5p and (*right*) miR-29c-3p for predicting PDAC (n=26) vs. benign disease (n=10) in plasma. AUC: area under the curve; EV: extracellular vesicle; PDAC: pancreatic ductal adenocarcinoma; ROC: receiver operating characteristic.

**
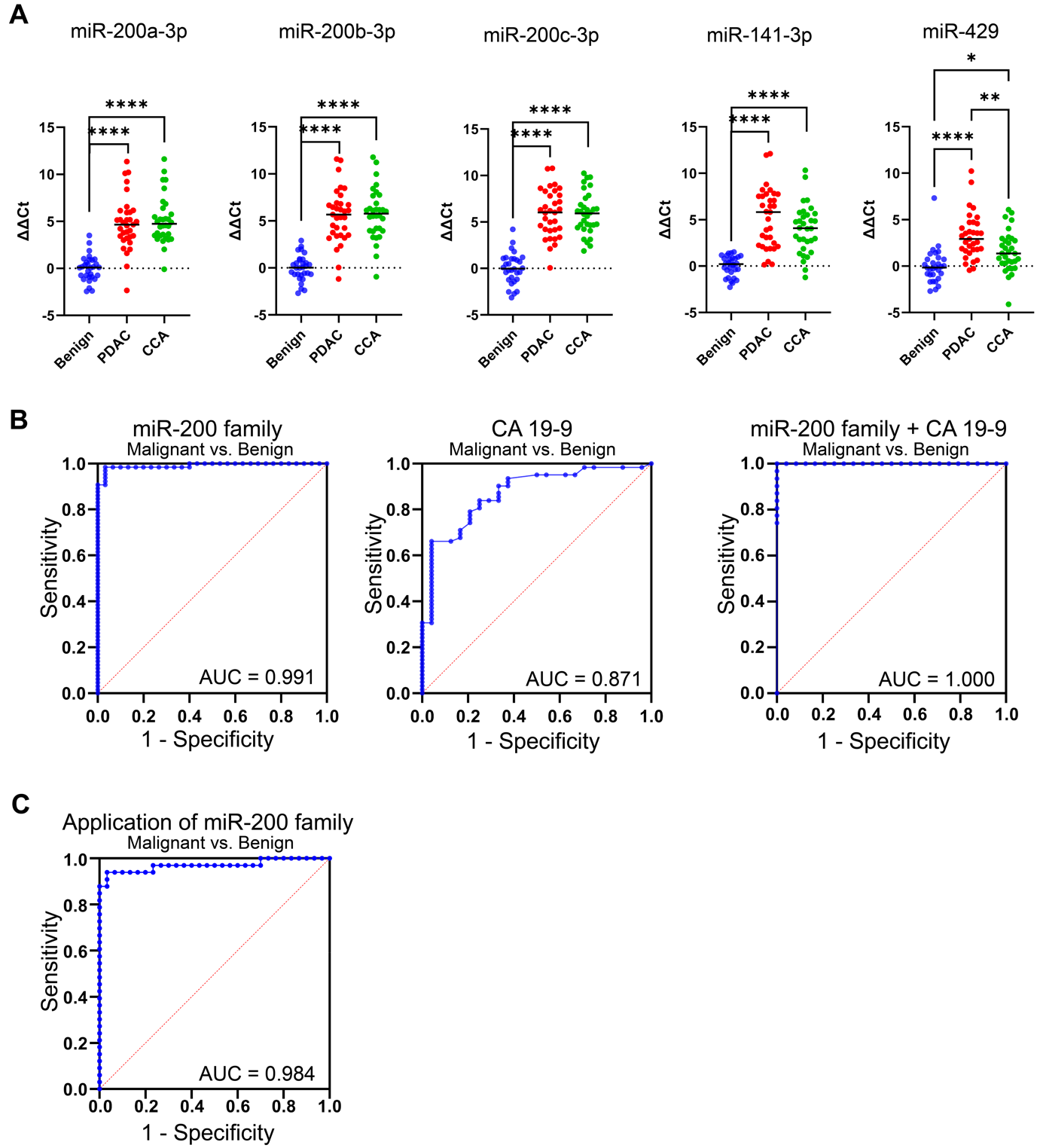
**

**Supplementary Figure 5. Differential expression of the miR-200 family members and application of the model in an independent cohort with benign and malignant disease (PDAC and CCA).** (**A**) RT-qPCR results of the miR-200 family (miR-200a-3p, miR-200b-3p, miR200c-3p, miR-141-3p, miR-429) in the clinical validation cohort (30 benign disease, 33 PDAC and 32 CCA). (**B**) ROC curves and corresponding AUCs for (*left)* the miR-200 family, (*middle*) CA 19-9, and (*right*) the combination of CA 19-9 and the miR-200 family. (**C**) The ability of the EV-miR-200 family model to predict malignant disease (vs. benign disease) when applied to the clinical validation cohort. AUC: area under the curve values; PDAC: pancreatic ductal adenocarcinoma; ROC: receiver operating characteristic. **p*<0.05, ***p*<0.005, ****p*<0.0005, *****p*<0.0001

**
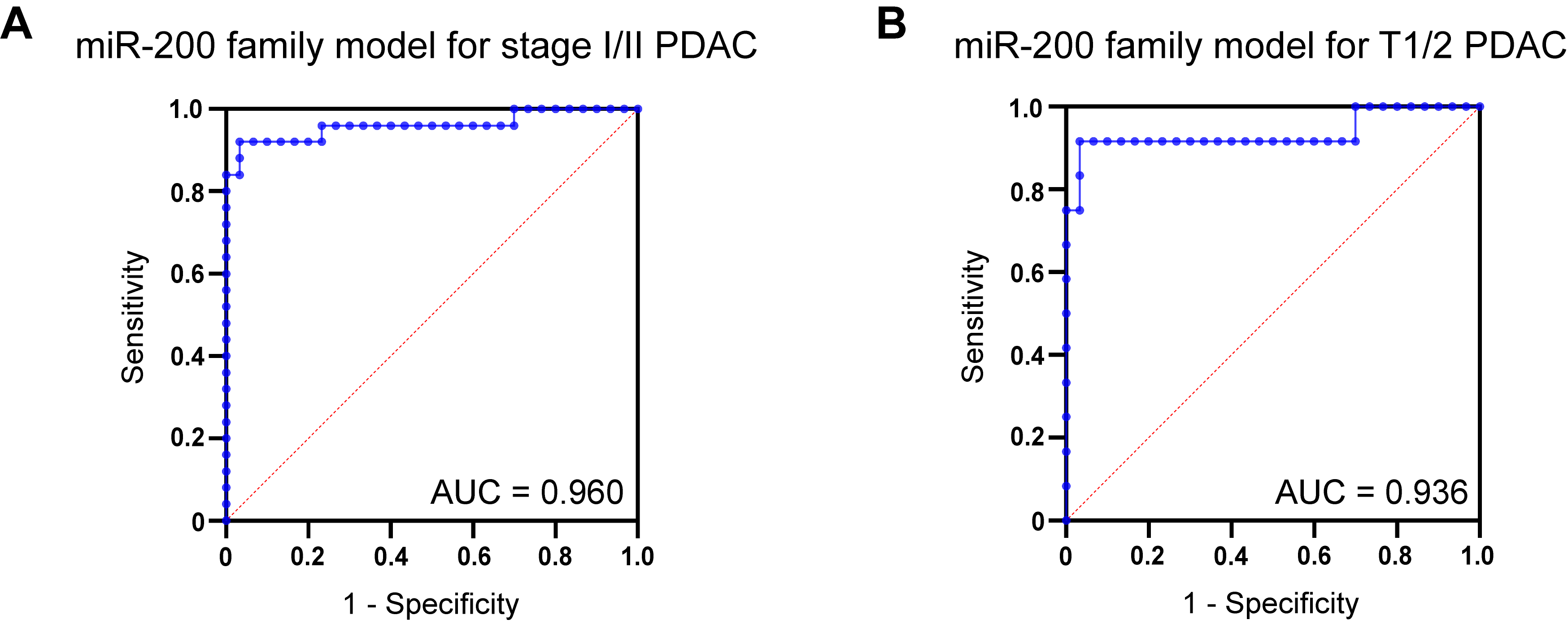
Supplementary Figure 6. Application of the plasma miR-200 family model to early-stage PDAC.** (**A**) Application of the plasma EV-miR-200 family model to early-stage PDAC (stage I/II, n=25) vs. benign (n=30) disease generated an AUC of 0.960 (95%CI 0.902-1.00; *p*<0.0001). (**B**) Application of the model to the subset of stage I/II PDAC with T1 or T2 (n=12) vs. benign disease (n=30) generated an AUC of 0.936 (95%CI 0.825-1.00; *p*<0.0001).

| **Table S1** Cycle threshold (Ct) values* of healthy donors. | | | | | | | |
| --- | --- | --- | --- | --- | --- | --- | --- |
| **Nr** | **Sex** | **Age^#^** | **miR-200a** | **miR-200b** | **miR-200c** | **miR-141** | **miR-429** |
| HD1 | F | 33 | 45 | 44 | 41 | 43 | 44 |
| HD2 | F | 45 | 44 | 43 | 42 | 41 | 44 |
| HD3 | F | 53 | 43 | 45 | 45 | 42 | 43 |
| HD4 | M | 54 | 44 | 44 | 43 | 44 | 44 |
| HD5 | M | 61 | 44 | 44 | 43 | 43 | 43 |
| HD6 | M | 63 | 44 | 44 | 45 | 43 | 45 |
| HD7 | F | 60 | 44 | 44 | 42 | 42 | 44 |
| HD8 | M | 47 | 43 | 42 | 45 | 44 | 45 |
| HD9 | M | 25 | 45 | 43 | 44 | 43 | 43 |
| HD10 | F | 55 | 43 | 45 | 42 | 44 | 45 |
| HD11 | M | 47 | 45 | 45 | 45 | 45 | 44 |
| HD12 | F | 48 | 44 | 43 | 44 | 43 | 43 |
| HD13 | M | 50 | 44 | 42 | 39 | 43 | 44 |
| HD14 | M | 38 | 43 | 42 | 41 | 44 | 44 |
| *Each Ct value shown is the averages of three technical replicates. #Age at time of venipuncture. Mir-23, miR-26a and UniSP6 were used as normalizer. | | | | | | | |
